# Promoting Hand Hygiene During the COVID-19 Pandemic: Randomized Controlled Trial of the Optimized Soapp+ App

**DOI:** 10.2196/57191

**Published:** 2025-04-24

**Authors:** Dario Baretta, Carole Lynn Rüttimann, Melanie Alexandra Amrein, Jennifer Inauen

**Affiliations:** 1 Institute of Psychology University of Bern Bern Switzerland

**Keywords:** COVID-19, hand hygiene, behavior change technique, Multiphase Optimization Strategy, randomized controlled trial, smartphone apps, mobile phones

## Abstract

**Background:**

The adoption of protective behaviors represents a crucial measure to counter the spread of infectious diseases. The development of effective behavior change techniques therefore emerged as a public health priority during the COVID-19 pandemic, but randomized controlled trials (RCTs) testing such interventions during the pandemic were scarce. We conducted a Multiphase Optimization Strategy to develop, optimize, and evaluate a smartphone app, Soapp+, to promote hand hygiene during the COVID-19 pandemic.

**Objective:**

This RCT aims to evaluate the efficacy of the Soapp+ app (intervention group) targeting motivation and habit compared to a simplified version of the app mainly delivering hand hygiene information (active control group). We hypothesize that, compared to the control group, the intervention group will show greater improvements in hand hygiene behavior and behavioral determinants post intervention and at a 6-month follow-up.

**Methods:**

We conducted an RCT from March 2022 to April 2023, recruiting 193 adults living in Switzerland online. Following baseline assessment, the intervention lasted 32 days, followed by a postintervention assessment and a 6-month follow-up. The primary outcome was the change in hand hygiene behavior from pre- to postintervention and preintervention to follow-up. Hand hygiene was assessed with electronic diaries. The intervention group received content incorporating various behavior change techniques designed to address key motivational and volitional determinants of hand hygiene behavior (eg, skills, knowledge, intention, attitudes toward hand hygiene, risk perception, outcome expectancies, self-efficacy, action planning, coping planning, action control, habit). In contrast, the active control group was exposed to behavior change techniques targeting only a subset of these determinants (ie, skills, knowledge, and intention). The delivery of the intervention content was fully automated. Group differences were tested using an intention-to-treat approach with the nonparametric Wilcoxon rank sum test.

**Results:**

Of the 193 randomized participants, 146 completed the first hand hygiene diary preintervention and were included in the main analysis. The mean age was 41 (SD 17) years, and 69.2% (n=101) were women. The main analysis revealed significant superiority of the intervention compared to controls in the change in hand hygiene pre-post intervention (W=2034; *P*<.04; effect size *r*=0.17) and between preintervention and follow-up (W=2005; *P*<.03; effect size *r*=0.18). Regarding behavioral determinants, the change in coping planning pre-post intervention (W=3840; *P*=.03, effect size *r*=0.16) was significantly greater in the intervention group using Soapp+ compared to controls.

**Conclusions:**

Soapp+ was developed through a rigorous experimental method during the ongoing COVID-19 pandemic. The RCT provided evidence for the efficacy of Soapp+ to promote hand hygiene in the context of a pandemic.

**Trial Registration:**

ClinicalTrials.gov NCT04830761; https://clinicaltrials.gov/study/NCT04830761

## Introduction

### Background

The COVID-19 pandemic represented an exceptional threat to human health worldwide. In that context, the collective adoption of protective behaviors (eg, hand hygiene, physical distancing, mask wearing) became an essential measure to prevent the transmission of the virus, especially during the pandemic outbreak when there was no vaccine available [1,2]. Consequently, the development of effective behavior change techniques aiming at supporting the uptake and adoption of such behaviors emerged as a public health priority [3]. However, during the course of the outbreak, there was limited or no contextualized knowledge about how to effectively promote protective behaviors in the general population [4,5].

During the early stages of the pandemic, we addressed the call for applying principles of behavior change to reduce the transmission of COVID-19 [2,3] and devised a Multiphase Optimization Strategy (MOST) [6] to develop, optimize, and evaluate a smartphone-based behavior change technique to promote hand hygiene during the COVID-19 pandemic [7,8]. We opted for a digital intervention to avoid in-person interaction, which is crucial for preventing virus transmission. Additionally, digital interventions can be tailored to individual needs and have the potential to reach a large number of users. The MOST framework was deemed highly suitable for rigorously contextualizing the intervention to the pandemic through optimization. We targeted correct hand hygiene (ie, hand washing or sanitizing) at key times because it represents an effective strategy in preventing the transmission of respiratory illnesses, including COVID-19 [9,10], and was therefore included in public health guidelines [11].

Following the MOST framework [12], the project included 3 phases: preparation, optimization, and evaluation. In the preparation phase [7], we developed a set of intervention modules and defined the optimization criteria. The development of intervention modules was guided by the Theoretical Domain Framework [13]. First, we identified behavioral determinants of hand hygiene through a literature review, and through 2 focus group discussions with the target population [7]. Then, we mapped behavior change techniques [14] to the behavioral determinants. Specifically, three intervention modules were developed targeting (1) motivation, (2) habit formation, and (3) social influence. The identified modules were paired and sequenced, resulting in 9 different intervention conditions. Each intervention condition was hosted in the developed smartphone app Soapp. During the subsequent optimization phase, we devised a mixed method double-blind parallel randomized trial between March 2021 and August 2021, which lasted 34 days. The trial aimed at identifying the intervention condition that met our optimization criteria: superiority regarding change in hand hygiene pre-post intervention, and regarding user satisfaction, usability, and engagement [8]. The results of the trial showed that hand hygiene improved while using the Soapp app. However, no superiority was found for any of the versions of the app. The qualitative results indicated that participants preferred the motivation and habit formation modules over the social influence ones. A further emerging theme pointed out the need for better distribution of intervention content over time. Thus, in the optimized version of the Soapp app, named Soapp+, we selected the motivation and habit formation modules and delivered them in a parallel fashion rather than sequentially.

Soapp+ comprises behavior change techniques aimed at targeting key motivational and volitional behavioral determinants including intention, attitudes toward hand hygiene, risk perception, outcome expectancies, self-efficacy, action planning, coping planning, action control, and habit. These determinants represent fundamental psychological constructs in prominent behavior change theories [15], addressing both reflective and automatic processes. Reflective processes are deliberate and conscious, involving setting intended goals, planning, and monitoring progress [16,17]. In contrast, automatic processes, such as habit, operate unconsciously, enabling individuals to perform behaviors without deliberate thought once learned through repetition. These automatic processes help sustain behavior change by reducing the cognitive load associated with decision-making and promoting consistency in behavior performance [18,19].

### Aims

The current paper focuses on the evaluation phase of the MOST in which we tested the efficacy of Soapp+ (intervention group) by comparison to an active control group in a randomized controlled trial (RCT). As presented in the study protocol [7], we tested the following preregistered hypotheses

- H1: The intervention group shows a greater increase in correct hand hygiene behavior at key times at the postmeasure (H1a) and at the 6-month follow-up (H1b) than the control group.
- H2: The intervention group shows a significant increase in the targeted behavioral determinants compared with the control group at the postintervention measure (H2a) and at the 6-month follow-up (H2b). Specifically, the target behavioral determinants were intention, attitude, risk perception, outcome expectancies, self-efficacy, action planning, coping planning, action control, and habit strength.

As the secondary aim, we examined between-group differences in flu-like infection symptoms and occurrences of COVID-19 (secondary outcomes). The corresponding results are reported in Multimedia Appendix 1.

## Methods

### Study Design

The study design for the evaluation phase was a double-blind RCT (1:1 ratio) comparing the optimized app Soapp+ with an active control group using a simplified version of the app that focused on providing information on hand hygiene. The trial was carried out from March 9, 2022, to April 18, 2023, a few days before the World Health Organization declared the end of the global health emergency [20].

### Participants

The target population was German-speaking adults from the Swiss population interested in using an app to improve hand hygiene. Inclusion criteria were (1) being at least 18 years of age, (2) owning a smartphone with mobile access to the internet, (3) being proficient in the German language, and (4) having signed an informed consent form to participate in the study. Additionally, smartphone proficiency was an implicit eligibility criterion, as participants needed to perform various tasks on their smartphones prior to the study’s commencement (eg, downloading the app and registering). As per protocol [7], the initial target sample size was 205 participants. The sample size was calculated a priori for an independent sample 1-tailed *t* test (β=.80; α=.05; Cohen *d*=0.35). The target sample size was raised to 245 participants to account for a 20% dropout rate.

### Measures

#### Primary Outcome

The frequency of correct hand hygiene at key times was assessed using ecological momentary assessment with an electronic diary embedded in the study apps (ie, Soapp+ and active control apps). This approach was used to avoid retrospective bias in reporting hand hygiene [21]. On study days 2, 8, 16, 24, and 32, participants were prompted 5 times per day to indicate whether each of the 13 key times to perform hand hygiene defined by the Swiss Federal Office of Public Health occurred (eg, arriving home, after using the toilet; Table S2 in Multimedia Appendix 1). For each key situation that occurred, participants were asked how often they correctly washed or disinfected their hands. The 5-point response scale ranged from 0=never to 4=always. For each diary day, the hand hygiene score was calculated by (1) computing the mean frequency of correct hand hygiene across all indicated key times in a single diary and then (2) averaging the hand hygiene frequency across the five diaries.

#### Secondary Outcomes

##### Targeted Behavioral Determinants

The following behavioral determinants were assessed at T1, T2, and the follow-up. These variables were selected based on the results of the optimization phase and represented the determinants targeted by the Soapp+ intervention.

###### Behavioral Intention

One self-reported item was adapted from a study by Allan, Sniehotta, and Johnston [22] asked “To what extent do you intend to correctly perform your hand hygiene behavior at key times?” The response options ranged from 1=not at all to 6=very strongly.

###### Action Planning

This was assessed using the mean score of 3 self-reported items [23], such as “I have made a detailed plan about when I am going to wash and disinfect my hands.” The response options ranged from 1=not at all to 6=very strongly. The scale was reliable (Cronbach α=0.90 at T1).

###### Coping Planning

This was assessed using the mean score of 4 self-reported items, such as “I have made a detailed plan about how I can perform correct hand hygiene behavior at key times when soap and water are not available.” The response options ranged from 1=not at all to 6=very strongly (Cronbach α=0.84 at T1).

###### Habit Strength

This was assessed with the “Self-Report Behavioural Automaticity Index” [24] with 4 items, such as “Correct hand hygiene behavior at key times is something that I do automatically.” The response options ranged from 1=strongly disagree to 7=strongly agree (Cronbach α=0.93 at T1).

###### Action Control

This was assessed using the mean score of 3 self-reported items, such as “During the last two weeks I have constantly monitored myself whether I washed or disinfected my hands according to my plans.” The response options were adapted from a study by Sniehotta, Scholz, and Schwarzer [25] and ranged from 1=not at all to 6=very strongly (Cronbach α=0.91 at T1).

###### Self-Efficacy

This was assessed using the mean score of 8 self-reported items, such as “How confident are you that you correctly wash/disinfect your hands at key times, when you are on the way.” The response options ranged from 1=not at all to 6=very strongly. Self-efficacy items were adapted from a study by Schwarzer [23] (Cronbach α=0.91 at T1).

###### Attitude

This was assessed using the mean score of 6 self-reported bipolar items [26] introduced by the wording “Correct hand hygiene behavior at key times is…” and followed by pairs of bipolar adjectives (eg, useless vs useful; Cronbach α=0.81 at T1).

###### Outcome Expectancies

This was assessed using the mean score of 8 self-reported items [27], such as “If I engage in correct hand hygiene, then then the risk drops that I contract pathogens.” The response options ranged from 1=this doesn’t apply at all to 6=this applies very precisely (Cronbach α=0.72 at T1).

###### Risk Perception

This was assessed using the mean score of 5 self-reported items [27], such as “to what extent do you think that you could contract corona, if you don't engage in correct hand hygiene in key situations?” The response options ranged from 1=this doesn’t apply at all to 6=this applies very precisely (Cronbach α=0.90 at T1).

##### Flu-Like Infection Symptoms and Occurrences of COVID-19

Participants reported on these secondary outcomes by answering the items “In the last two weeks, did you have flu-like infection symptoms?” and “In the last two weeks, did you have a positive corona test?”

Finally, social desirability was assessed at T1 using the German Short Scale for the two-dimensional measurement of social desirability [28].

### Procedure

Participants were recruited via social media (eg, Facebook, Instagram), mailing lists, and leaflets with the support of a market research company. We aimed at recruiting a broad range of people from the German-speaking adult Swiss general population in terms of sex, age, and socioeconomic status. The content of the recruitment materials targeted individuals interested in forming new hand-hygiene habits and learning how to correctly wash or disinfect their hands to counter the spread of the virus. Interested people were redirected to the study page on RedCap (Vanderbilt University) [29] where they could go through the study information, fill out an eligibility and consent survey, and sign the e-consent form. Afterward, participants received a registration code via email and were guided to download fthe Soapp+ app from the Apple App Store or Google Play Store and register it. The day after the registration, participants received the baseline questionnaire (T1) and were then randomized to an intervention or active control group. A simple randomization was implemented in Qualtrics, which preserved the allocation concealment. Additionally, the researchers involved in the study were blinded to intervention assignment because the participant identifier was pseudoanonymized before randomization (Figure S1 in Multimedia Appendix 1). During the intervention phase, participants completed the hand hygiene diary on days 2, 8, 16, 24, and 32. At the end of the intervention phase (day 34), participants received a second questionnaire (T2). At 6 months (182 days) after T1, participants received the last follow-up questionnaire. Questionnaires and diaries were integrated into Qualtrics services using Soapp+’s application programming interface, and the participants’ data were stored on Qualtrics. The recruitment for the evaluation trial began on March 9, 2022, and ended on October 20, 2022. Follow-up data were collected between September 5, 2022, and April 18, 2023.

### Intervention

The content of the intervention was delivered to participants via their personal smartphone through the study app Soapp+. The intervention group received the optimized Soapp+ app while the active control group was exposed to a simplified version of Soapp+. Table 1 shows the content delivered. The exact wording of the intervention messages and the intervention timeline can be found in Table S3 and Figure S2 in Multimedia Appendix 1.

**Table 1:** Intervention content overview.

Condition and module			TDF^a^ domain		Behavioral determinant		Behavior change technique
**Intervention and active control groups**							
	Basic	Goals		Intention		1.1 Goal setting (behavior)	
	Basic	Skills		Skills		4.1 Instruction on how to perform behavior	
	Basic	Knowledge		Knowledge		5.1 Information about health consequences	
**Active control group**							
	Quizzes	Knowledge		Knowledge		5.1 Information about health consequences	
	Quizzes	Skills		Skills		4.1 Instruction on how to perform behavior	
**Intervention group**							
	Motivation	Goals		Intention		1.1 Goal setting (behavior)	
	Motivation	Beliefs about consequences		Risk perception		5.1 Information about health consequences	
	Motivation	Beliefs about consequences		Attitude		5.2 Salience of consequences	
	Motivation	Beliefs about consequences		Outcome expectancies		9.2 Pros and cons	
	Motivation	Beliefs about consequences		Intention		5.2 Salience of consequences	
	Motivation	Beliefs about capabilities		Self-efficacy		1.2 Problem-solving 15.1 Verbal persuasion about capabilities 15.3 Focus on past success	
	Motivation	Beliefs about capabilities		Coping planning		1.2 Problem solving	
	Motivation	Reinforcement		Intention		10.9 Self-reward	
	Habit	Knowledge		Knowledge		4.2. Information about antecedents	
	Habit	Memory, attention, and decision processes		Action control		2.3 Self-monitoring of behavior	
	Habit	Goals		Action planning		1.4 Action planning 7.1. Prompts or cues	
	Habit	Skills and goals		Habit strength		8.1 Behavioral practice or rehearsal 8.3 Habit formation	
	Habit	Behavioral regulation		Habit strength		7.1 Prompts or cues (physical cue)	

^a^TDF: theoretical domains framework [29].

### Active Control Group

The active control group received information on how to perform correct hand hygiene (both in written and video formats), the key times to perform hand hygiene, and a statement of the goal of the app (basic module). This content was accessible throughout the intervention phase. Based on the results of the optimization phase, we also added a list of fun facts that were delivered to the participants via push notifications throughout the intervention phase. Finally, quizzes on hand hygiene aimed at ensuring that the active control group interacted with the app throughout the intervention phase.

### Intervention Group

Like the control group, the intervention group received the basic module. They additionally received the content targeting motivation and habit. Based on the results of the optimization phase (ie, request for better-distributed content or tasks), this content was delivered in parallel instead of sequentially as done during the optimization trial.

### Data Analysis

#### Handling of Missing Data

To handle missing data, the intention-to-treat (ITT) principle was adopted [30]. According to the ITT principle, all the randomized participants are included in the analysis, regardless of what happens after randomization (eg, noncompliance, protocol deviations). Therefore, the ITT approach avoids overoptimistic estimates of the efficacy of an intervention resulting from the removal of noncompliers. Missing data and dropouts were addressed using the last observation carried forward approach. The last observation carried forward approach represents a parsimonious yet effective method to maintain appropriate type 1 error protection [31]. This approach has been used in previous behavior change research [32], including the optimization trial of the *Soapp* app [8]. This methodological choice was reinforced by the results of a secondary analysis conducted on the optimization trial [33]. The analysis revealed a noteworthy intraclass correlation of 0.60 in hand hygiene, signifying that a substantial portion of the observed behavior can be attributed to individual differences between persons. This implies that the behavior tends to be more consistent or less variable within individuals over time. Finally, we also conducted a set of sensitivity analyses without missing value imputation; results are available in Multimedia Appendix 1.

#### Hypothesis Testing

In order to test the hypotheses, the assessment points considered for hand hygiene behavior were the first diary on day 2 (T1), the diary day on day 32 (T2, postintervention), and the last diary day on day 181 (follow-up). The difference in hand hygiene between T2 and T1 and follow-up and T1 was compared between the intervention and the active control groups using the nonparametric Wilcoxon rank sum test. A positive value in hand hygiene difference over assessments indicated an increase in hand hygiene. Likewise, positive values in the targeted behavioral determinants over assessments indicated an increasing trend. The Wilcoxon rank sum test was chosen over the preregistered 1-tailed Student *t* test due to the nonnormal distribution of the data, as assessed by the Shapiro-Wilk normality test, representing a deviation from the registered protocol. Results of the analysis with the Student *t* test are available in Table S4 in Multimedia Appendix 1.

#### Analytical Software

The Soapp+ app was developed by the study authors using technological resources provided by the Faculty of Human Sciences at the University of Bern, with support from an internal software engineer. The statistical software R (version 4.3.0; R Foundation for Statistical Computing) was used to process the data and run the analyses. The data and R code used for the main analyses are available on the Open Science Framework repository platform [34].

### Ethical Considerations

The trial received ethical approval from the Swiss Ethics Committee of the canton of Bern (ID 2021-00164). The reporting of the trial is in line with the CONSORT (Consolidated Standards of Reporting Trials) guidelines (Multimedia Appendix 2) [35]. Informed consent was obtained from all participants, who were also informed of their right to opt out at any stage of the study. To protect privacy and confidentiality, data were deidentified through pseudonymization. As compensation, participants had the opportunity to win one of three iPhone 12s as an incentive for their participation. Additionally, after completing T2, participants were offered a small gift (ie, a bar of hand soap and a thank you card) in order to prevent dropout, which was sent to their homes.

## Results

### Overview

**Figure 1:**
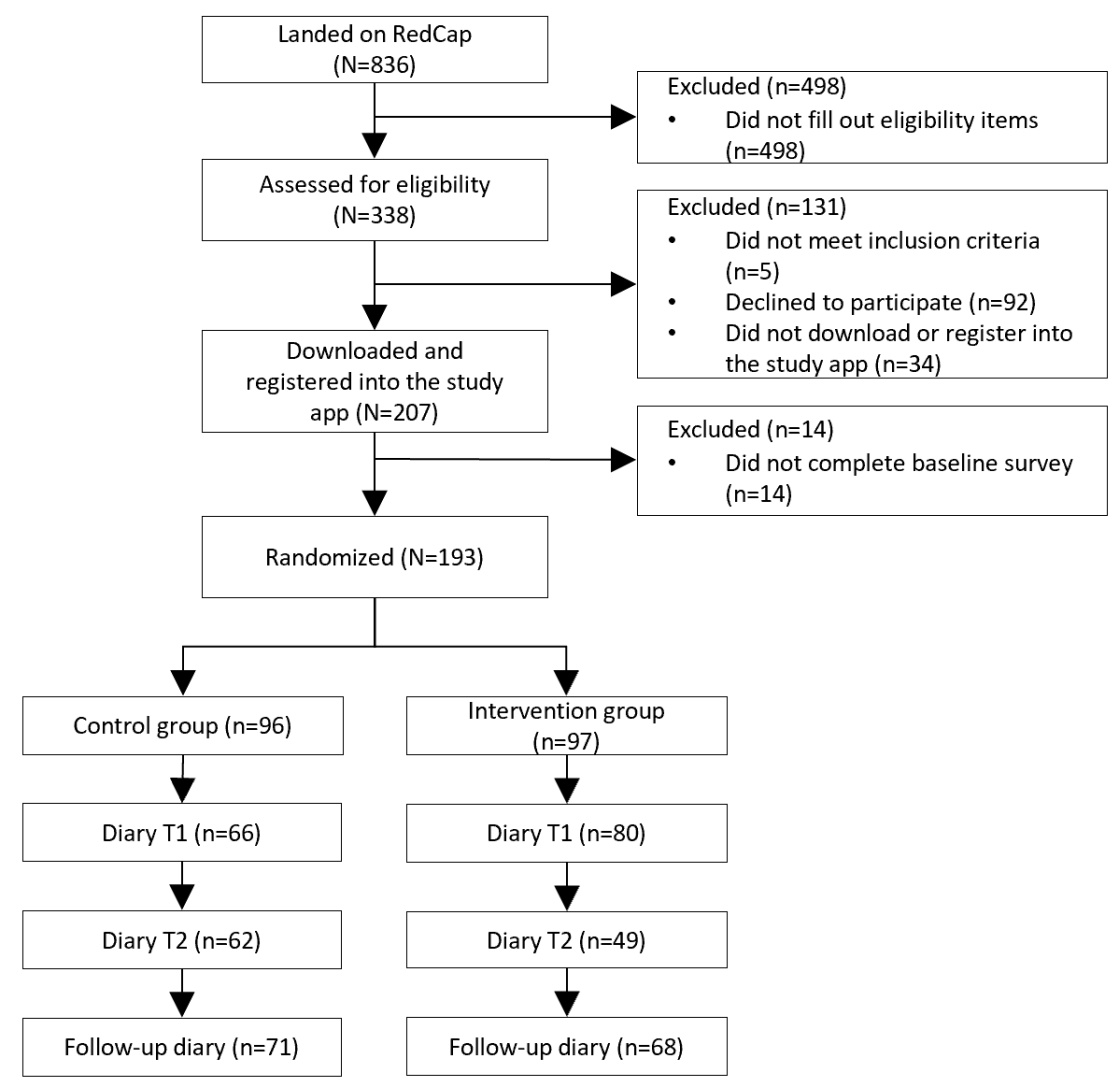
Participant recruitment flow.

We stopped the trial 13 months after the start of the study due to the end of the project timeline. A total of 193 participants were recruited and randomized into one of the 2 experimental conditions. Among these, 12 participants did not complete any of the 6 hand hygiene diary days, while the other 35 participants did not fill out the first diary at T1. Further, 3 participants completed the first diary but did not encounter any of the key situations to perform hand hygiene during that day. These participants (n=50) were excluded from the analysis because the main outcome (ie, hand hygiene) at T1 was missing. Out of the 193 participants who were randomized, 146 (75.6%) filled out the hand hygiene diary at T1, 91 of these (47.2% of the randomized participants) completed the hand hygiene diary at T2, and 109 (56.5% of the randomized participants) completed the hand hygiene diary at the 6-month follow-up. Figure 1 shows the participants’ flow through randomization, T1 diary assessment, T3 diary assessment, and follow-up for each intervention group.

### Baseline Characteristics

Sociodemographics and hand hygiene behavior at baseline are reported in Table 2 (see Table S1 in Multimedia Appendix 1 for a comparison with the sociodemographic characteristics of the Swiss population). The figures refer to the 146 participants who completed the first diary at T1. Participants’ mean age was 41 (SD 17) years, 69.2% (n=101) were women, 69.9% (n=102) had high school qualifications, 45.9% (n=67) were employed, and 24% (n=35) were living alone. Descriptive statistics in hand hygiene behavior (mean 3.05, SD 0.78; median 3.25, IQR 2.63-3.60; skewness=–1.24) suggested that hand hygiene behavior was already high at baseline and characterized by a moderate left-tailed distribution. No significant differences in social desirability were found between the intervention and the control group (M_1_=4.72; M_2_=4.79; *F*_1,189_=0.44; *P*=.51).

**Table 2:** Sociodemographic characteristics at baseline (T1; N=146).

Variable		Overall	Experimental group		*P* value^a^	
			Control (n=66)	Intervention n=80)		
Age (years), mean (SD)		41 (17)	42 (17)	40 (17)	.56	
**Sex, n (%)**						.49
	Diverse	3 (2.1)	1 (1.5)	2 (2.5)		
	Male	42 (28.8)	16 (24)	26 (33)		
	Female	101 (69.2)	49 (74)	52 (65)		
**Marital status, n (%)**						.01
	Divorced	12 (8.2)	8 (12)	4 (5)		
	Single	88 (60.3)	32 (48)	56 (70)		
	Married	44 (30.1)	26 (39)	18 (23)		
	Widowed	2 (1.4)	0 (0)	2 (2.5)		
**Have children, n (%)**						.047
	Yes	43 (29.5)	25 (38)	18 (23)		
	No	103 (70.5)	41 (62)	62 (78)		
**Education, n (%)**						.15
	High school diploma	102 (69.9)	42 (64)	60 (75)		
	Completed primary school	2 (1.4)	2 (3)	0 (0)		
	Completed secondary school	42 (28.8)	22 (33)	20 (25)		
**Higher education, n (%)**						.20
	Completed vocational training (apprenticeship)	35 (24)	21 (32)	14 (18)		
	Completed (applied) university degree	59 (40.4)	26 (39)	33 (41)		
	Other educational or vocational qualification	14 (9.6)	5 (7.6)	9 (11)		
	No educational or vocational training	38 (26)	14 (21)	24 (30)		
**Profession, n (%)**						.59
	Unemployed	4 (2.7)	1 (1.5)	3 (3.8)		
	Employed	67 (45.9)	35 (53)	32 (40)		
	Homemaker	8 (5.5)	4 (6.1)	4 (5)		
	In training or retraining	2 (1.4)	1 (1.5)	1 (1.3)		
	Retired	18 (12.3)	8 (12)	10 (13)		
	Student	47 (32.2)	17 (26)	30 (38)		
**Monthly income^b^, n (%)**						.25
	Less than 2000 Fr	11 (7.5)	3 (4.5)	8 (10)		
	Between 2000 and 4000 Fr	17 (11.6)	12 (18)	5 (6.3)		
	Between 4001 and 6000 Fr	29 (19.9)	13 (20)	16 (20)		
	Between 6001 and 8000 Fr	23 (15.8)	7 (11)	16 (20)		
	Between 8001 and 10,000 Fr	23 (15.8)	11 (17)	12 (15)		
	More than 10,000 Fr	26 (17.8)	12 (18)	14 (18)		
	I don’t know	17 (11.6)	8 (12)	9 (11)		
**Living situation, n (%)**						.42
	Living by themselves	35 (24)	15 (23)	20 (25)		
	Living with family	67 (45.9)	35 (53)	32 (40)		
	Living in a shared apartment	33 (22.6)	12 (18)	21 (26)		
	Other	11 (7.5)	4 (6.1)	7 (8.8)		

^a^Welch 2-sided *t* test or Fisher exact test.

^b^Swiss francs. When the study started in March 2022, the average conversion rate between Swiss francs and US dollars was 0.93, meaning that US $1 was equal to 0.93 Swiss francs

### Dropout Analysis

Dropout analysis was performed to investigate baseline differences between participants who reached the end of the intervention phase and those who dropped out at any point during the intervention. We analyzed all 193 randomized participants and the ones who did not complete the T2 panel assessment were categorized as dropouts (n=120). The results suggested that participants who dropped out were significantly younger than retainers (M_1_=39; M_2_=46; *F*_1, 191_=7.06; *P*=.009). Dropouts and retainers did not differ regarding sex (male vs female; *χ*^2^_1_=1.72; *P*=.19), hand hygiene (*F*_1,190_=0.12; *P*=.73), intention to increase hand hygiene behavior (*F*_1,191_=2.36; *P*=.13), or intervention group allocation (*χ*^2^_1_=2.04; *P*=.15).

### Hand Hygiene Behavior

Hand hygiene behavior at T1, T2, and follow-up is summarized in Table 3. The whole sample exhibited a nonsignificant increase in hand hygiene behavior from T1 (median 3.25, IQR 2.63-3.60) to T2 (median 3.42, IQR 2.62-3.88; *W*=8904; *P*=.06, *r*=0.11). This was followed by a significant decrease between T2 and the 6-month follow-up (median 3.25, IQR 2.50-3.65; *W*=11,655; *P*=.04; *r*=0.12). When looking at changes in hand hygiene within each experimental group, the data shows that hand hygiene between T1 and T2 remained almost stable for the active control group (*W*=2050; *P*=.77; *r*=0.03), while the intervention group showed a significant increase in hand hygiene after the intervention phase (*W*=2386; *P*=.02; *r*=0.19). At follow-up, both the active control group (*W*=2313; *P*=.35; *r*=0.08) and the intervention group (*W*=2924; *P*=.68; *r*=0.03) exhibited a nonsignificant decrease in hand hygiene compared to T1.

The between-group differences in hand hygiene behavior between T1 and T2 and T1 and follow-up are reported in Table 4. Nonparametric analysis with the Wilcoxon rank sum test showed that the change in hand hygiene behavior between T1 and T2 (*W*=2034; *P*<.04; effect size *r*=0.17) and between T1 and follow-up (*W*=2005; *P*<.03; effect size *r*=0.18) significantly differed between the groups, indicating better hand hygiene in the intervention group.

**Table 3:** Hand hygiene and targeted behavioral determinants median values at each assessment point and Shapiro-Wilk normality test.

Variable and assessment point				Participants, n		Overall, median (IQR)		Active control group, median (IQR)		Intervention group, median (IQR)		Shapiro-Wilk normality test		
												Statistic		*P* value
**Target behavior**														
	**Hand hygiene**													
		T1	143		3.25 (2.63-3.60)		3.23 (2.56-3.57)		3.25 (2.71-3.61)		0.897		<.001	
		T2	143		3.42 (2.62-3.88)		3.29 (2.55-3.71)		3.58 (3.00-3.94)		0.852		<.001	
		Follow-up	143		3.25 (2.50-3.65)		3.14 (2.33-3.55)		3.29 (2.57-3.77)		0.894		<.001	
**Behavioral determinants**														
	**Intention**													
		T1	193		5.00 (5.00-6.00)		5.00 (5.00-5.25)		5.00 (5.00-6.00)		0.823		<.001	
		T2	193		5.00 (5.00-6.00)		5.00 (5.00-6.00)		5.00 (5.00-6.00)		0.796		<.001	
		Follow-up	193		.00 (4.00-6.00)		5.00 (4.00-5.00)		5.00 (4.00-6.00)		0.841		<.001	
	**Action planning**													
		T1	193		4.33 (4.00-5.00)		4.33 (3.67-5.00)		4.67 (4.00-5.00)		0.922		<.001	
		T2	193		4.67 (4.00-5.00)		4.67 (4.00-5.00)		5.00 (4.00-5.00)		0.890		<.001	
		Follow-up	193		4.67 (3.67-5.00)		4.33 (3.67-5.00)		4.67 (3.67-5.00)		0.916		<.001	
	**Coping planning**													
		T1	193		3.75 (2.75-4.25)		3.50 (2.75-4.25)		3.75 (2.75-4.50)		0.974		.001	
		T2	193		4.00 (3.25-4.75)		3.75 (2.94-4.50)		4.25 (3.50-5.00)		0.971		<.001	
		Follow-up	193		4.00 (2.75-4.75)		3.75 (2.69-4.50)		4.00 (3.00-5.00)		0.966		<.001	
	**Habit strength**													
		T1	187		5.25 (4.00-6.25)		5.00 (3.50-6.00)		5.75 (4.19-6.25)		0.933		<.001	
		T2	187		5.25 (4.25-6.00)		5.25 (4.00-6.00)		5.25 (4.50-6.25)		0.940		<.001	
		Follow-up	187		5.25 (4.00-6.00)		5.00 (3.75-6.00)		5.25 (4.63-6.00)		0.932		<.001	
	**Action control**													
		T1	152		4.00 (3.33-5.00)		4.00 (3.58-5.00)		4.00 (3.33-5.00)		0.947		<.001	
		T2	152		4.67 (4.00-5.00)		4.33 (4.00-5.00)		4.67 (4.00-5.33)		0.938		<.001	
		Follow-up	152		4.33 (3.33-5.00)		4.33 (3.33-5.00)		4.33 (3.58-5.00)		0.955		<.001	
	**Self-efficacy**													
		T1	193		4.25 (3.63-4.88)		4.13 (3.50-4.75)		4.38 (3.63-5.00)		0.986		.049	
		T2	193		4.38 (3.75-5.00)		4.25 (3.75-4.88)		4.38 (3.88-5.00)		0.979		.005	
		Follow-up	193		4.25 (3.63-4.88)		4.13 (3.59-4.88)		4.25 (3.63-4.88)		0.984		.03	
	**Attitudes**													
		T1	188		5.17 (4.67-5.62)		5.17 (4.67-5.50)		5.17 (4.67-5.67)		0.932		<.001	
		T2	188		5.17 (4.67-5.50)		5.17 (4.50-5.50)		5.08 (4.67-5.67)		0.885		<.001	
		Follow-up	188		5.00 (4.33-5.50)		5.00 (4.46-5.38)		5.08 (4.33-5.67)		0.880		<.001	
	**Outcome expectancies**													
		T1	193		4.63 (4.25-5.13)		4.63 (4.25-5.00)		4.75 (4.25-5.13)		0.979		.005	
		T2	193		4.75 (4.25-5.13)		4.75 (4.25-5.13)		4.75 (4.25-5.13)		0.987		.08	
		Follow-up	193		4.63 (4.13-5.13)		4.63 (4.13-5.13)		4.63 (4.25-5.13)		0.987		.07	
	**Risk perception**													
		T1	193		4.60 (4.00-5.00)		4.60 (4.00-5.00)		4.60 (3.80-5.00)		0.942		<.001	
		T2	193		4.60 (4.00-5.00)		4.60 (4.00-5.00)		4.60 (4.00-5.00)		0.953		<.001	
		Follow-up	193		4.60 (3.60-5.00)		4.60 (3.60-5.00)		4.60 (3.80-5.00)		0.950		<.001	

**Table 4:** Differences in changes in hand hygiene and behavioral determinants between the intervention group and active control group with the Wilcoxon rank sum test.

Variables and change				Participants, n		Overall, median (IQR)		Active control group, median (IQR)		Intervention group, median (IQR)		*W* statistic		*P* value		Effect size *r*^a^
**Target behavior**																
	**Hand hygiene**															
		T1-T2	143		0.00 (–0.11 to 0.38)		0.00 (–0.23 to 0.35)		0.00 (0.00 to 0.41)		2034		.04		0.17	
		T1-Follow-up	143		0.00 (–0.41 to 0.23)		–0.04 (–0.52 to 0.05)		0.00 (–0.23 to 0.32)		2005		.03		0.18	
**Behavioral determinants**																
	**Intention**															
		T1-T2	193		0.00 (0.00 to 0.00)		0.00 (0.00 to 0.00)		0.00 (0.00 to 0.00)		4560		.75		0.02	
		T1-Follow-up	193		0.00 (0.00 to 0.00)		0.00 (0.00 to 0.00)		0.00 (–1.00 to 0.00)		4845		.57		0.04	
	**Action planning**															
		T1-T2	193		0.00 (0.00 to 0.33)		0.00 (0.00 to 0.33)		0.00 (0.00 to 0.33)		4395		.47		0.05	
		T1-Follow-up	193		0.00 (–0.33 to 0.33)		0.00 (0.00 to 0.33)		0.00 (–0.33 to 0.33)		4917		.49		0.05	
	**Coping planning**															
		T1-T2	193		0.00 (0.00 to 0.75)		0.00 (–0.25 to 0.50)		0.00 (0.00 to 1.00)		3840		.03		0.16	
		T1-Follow-up	193		0.00 (–0.25 to 0.75)		0.00 (–0.31 to 0.56)		0.00 (0.00 to 0.75)		4388		.48		0.05	
	**Habit strength**															
		T1-T2	187		0.00 (0.00 to 0.25)		0.00 (–0.13 to 0.25)		0.00 (0.00 to 0.25)		4585		.54		0.04	
		T1-Follow-up	187		0.00 (–0.50 to 0.38)		0.00 (–0.63 to 0.38)		0.00 (–0.31 to 0.31)		4469		.79		0.02	
	**Action control**															
		T1-T2	152		0.00 (0.00 to 0.67)		0.00 (0.00 to 0.67)		0.00 (0.00 to 0.42)		2996		.68		0.03	
		T1-Follow-up	152		0.00 (–0.33 to 0.33)		0.00 (–0.33 to 0.33)		0.00 (–0.33 to 0.08)		3075		.48		0.06	
	**Self-efficacy**															
		T1-T2	193		0.00 (0.00 to 0.25)		0.00 (0.00 to 0.25)		0.00 (0.00 to 0.25)		4824		.65		0.03	
		T1-Follow-up	193		0.00 (–0.38 to 0.25)		0.00 (–0.25 to 0.38)		0.00 (–0.38 to 0.25)		4881		.56		0.04	
	**Attitudes**															
		T1-T2	188		0.00 (0.00 to 0.00)		0.00 (0.00 to 0.17)		0.00 (0.00 to 0.00)		4650		.50		0.05	
		T1-Follow-up	188		0.00 (–0.33 to 0.17)		0.00 (–0.25 to 0.17)		0.00 (–0.33 to 0.00)		4747		.37		0.07	
	**Outcome expectancies**															
		T1-T2	193		0.00 (0.00 to 0.25)		0.00 (0.00 to 0.25)		0.00 (0.00 to 0.13)		5059		.28		0.08	
		T1-Follow-up	193		0.00 (–0.25 to 0.13)		0.00 (–0.25 to 0.25)		0.00 (–0.25 to 0.13)		5125		.22		0.09	
	**Risk perception**															
		T1-T2	193		0.00 (–0.20 to 0.00)		0.00 (–0.20 to 0.00)		0.00 (0.00 to 0.00)		4300		.33		0.07	
		T1-Follow-up	193		0.00 (–0.40 to 0.20)		0.00 (–0.60 to 0.20)		0.00 (–0.20 to 0.40)		3971		.07		0.13	

^a^The following guidelines are used to interpret the effect size (*r*): a large effect is defined as *r*≥0.50, a medium effect as approximately *r*=0.30, and a small effect as approximately *r*=0.10 [36].

### Targeted Behavioral Determinants

Behavioral determinants values at T1, T2, and follow-up are summarized in Table 3, while results from the paired Wilcoxon tests between T1 and T2 and T1 and follow-up are reported in Table S5 in Multimedia Appendix 1. Shapiro-Wilk normality test showed that almost all the behavioral determinants were not normally distributed at all assessment points, with the exception of outcome expectancies at T2 and follow-up (Table 3). The variables that increased the most in the whole sample between T1 and T2 were coping planning (*W*=1474; *P*<.001) and action control (*W*=611; *P*<.001), followed by action planning (*W*=1338; *P*=.006) and self-efficacy (*W*=2115; *P*=.01). The change in behavioral determinants between T1 and follow-up was less pronounced and sometimes negative (ie, intention, self-efficacy, attitudes, outcome expectancies, and risk perception). The between-group differences in the targeted behavioral determinants between T1 and T2 and T1 and follow-up are reported in Table 4. Results showed that the change in coping planning between T1 and T2 (*W*=3840*;* *P*=.03; effect size *r*=0.16) significantly differed between the intervention groups.

### Sensitivity Analysis

All the hypotheses were also tested without applying any missing value imputation algorithm. Results are available in Tables S6 and S7 in Multimedia Appendix 1 and confirm most of the findings. Additionally, Wilcoxon tests without missing values imputation showed between-group differences in coping planning between T1 and T2, and in risk perception between T1 and follow-up in favor of the intervention group.

## Discussion

### Efficacy of the Soapp+ App

As part of a MOST to develop, optimize, and test a smartphone-based hand hygiene intervention during the COVID-19 pandemic, this RCT aimed at evaluating the efficacy of the Soapp+ app intervention against an active control group. Results showed that hand hygiene can be increased short and long term using the Soapp+ app intervention compared to active controls toward the end of the COVID-19 pandemic. Specifically, we observed small but stable effect sizes (*r*=0.17 between pre-post intervention and *r*=0.18 between preintervention and follow-up) for an increase in hand hygiene among the intervention group compared to the control group. The results of this study contribute to the notion that simultaneously targeting both reflective and automatic processes is an effective strategy for promoting and sustaining behavior change, even in challenging contexts such as a pandemic.

Discussing the clinical relevance of the effect size is challenging due to the limited evidence available regarding the necessary target effect size for a hand hygiene intervention to prevent the acquisition or transmission of COVID-19 [37]. However, we can note that the effect sizes we observed were smaller than the average effect size typically reported in previous interventions, primarily RCTs, aimed at promoting hand hygiene to mitigate the transmission of respiratory viruses (Cohen *d*=0.62) [4]. One plausible reason for this difference is that the study’s target population consists of individuals who are already inclined toward hand hygiene behavior. This is evident from the fact that the frequency of hand hygiene in key situations was already relatively high in our sample at baseline, with median values of 3.25 and 3.23 out of 4 for the intervention and active control group, respectively, where a value of 3 indicates that hand hygiene was performed “often.” In spite of such a ceiling effect, participants in the intervention group still managed to increase the frequency of correct hand hygiene compared to those in the active control group.

Additionally, even though between-group differences in hand hygiene were small, we saw a trend that they resulted in fewer self-reported flu-like symptoms at postintervention and follow-up for the intervention group (Table S8 in Multimedia Appendix 1). Although this difference was not statistically significant due to the small sample size and claims about the clinical significance of the intervention cannot be made, it does indicate a tendency toward an intervention effect on flu-like symptoms. Finally, it should be noted that digital interventions like Soapp+ offer the advantage of scalability and the potential to reach a broader segment of the population. This is especially relevant in a pandemic context, where the diffusion of the virus’s transmission grows exponentially, and even small changes in individual behavior, enacted on a larger scale, have the potential to significantly contribute to countering the pandemic’s spread at the community level.

### Targeted Behavioral Determinants

Regarding our second hypothesis, results evidenced significant group differences pre-post intervention in coping planning in favor of the intervention group (Table 4). The fact that coping planning was the only behavioral determinant associated with group differences pre-post intervention aligns well with the intervention content targeting coping planning skills (ie, problem-solving) delivered toward the end of the intervention and close to the postintervention assessment (Multimedia Appendix 1). A possible explanation is that coping planning was a determinant with a low baseline value. Therefore, the intervention content might have played a significant role on this variable as there were more margins for improvement compared to the other targeted determinants.

### Strengths and Limitations

To the best of our knowledge (based on a search on ClinicalTrials.gov with the following key terms: condition or disease “COVID 19,” age “Adult (18-64),” study type “Interventional,” outcome measure “Hand hygiene,” this study is one of the few registered RCTs testing the effectiveness of a behavior change technique targeting hand hygiene in the general population during the COVID-19 pandemic. Additionally, this RCT study is the third of a series where we have used the MOST approach for intervention development. This feature of our project stands as a significant strength, given that the MOST places strong emphasis on a rigorous, evidence-based approach to developing, refining, and evaluating interventions. Moreover, the mixed but overall encouraging evidence for the efficacy of Soapp+ points to the potential value of this app as an effective tool to address the next pandemic. This aspect is of primary relevance, as before the pandemic, there was only limited or no contextualized knowledge about how to effectively promote hand hygiene during an outbreak [3,4]. In case of future epidemics, Soapp+ is a contextualized intervention that is readily available to be tested at a larger scale.

This study is not without limitations. First, the achieved sample size (N=143) was smaller than planned. Therefore, the probability of detecting a true effect of the intervention was below standard (ie, .80) and some small effect sizes might have been missed. The decision to stop the recruitment even though the target sample size was not reached was due to the project timeline [7], but also considering that the pandemic situation was developing toward better scenarios to the point where the World Health Organization declared the end of the global health emergency [20]. Based on the found effect size, future efficacy trials should be powered to detect small effects.

A second limitation is related to the dropouts between randomization and the first diary assessment. Indeed, the first diary was scheduled for the day after the randomization, and some participants who had been randomized (n=47) did not complete it. Other participants dropped out through the course of the study without filling out the assessment at T2 and follow-up. A possible explanation for the attrition might be due to the longitudinal study design with multiple diaries and other daily tasks that might have generated some fatigue. To address potential differences between dropouts and retainers, we conducted a dropout analysis, indicating a higher likelihood of younger participants dropping out. Thus, when extending these results to young adults, caution is advised.

Another limitation is the high prevalence of women in the trial (69%), resulting in a sex ratio that does not mirror the target population. This ratio aligns with the sample characteristics of the optimization trial [8] and other COVID-19 hand hygiene research [38]. This imbalance can be explained in light of empirical evidence suggesting that women were more worried about the COVID-19 pandemic and keener to adopt hand hygiene behavior [39]. Finally, the primary outcome of the study was self-reported, which can be biased by social desirability and recall issues [21]. The latter were minimized using the electronic hand hygiene diary. Nevertheless, social desirability might still have played a role.

### Implications, Challenges, and Considerations

A key practical implication is that the Soapp+ app, a behavior change technique contextualized to a pandemic, is now available and can be readily offered to the general public at the emergence of a future infectious disease outbreak (eg, via public health institutions). However, since the app’s efficacy has not yet been robustly demonstrated due to sample size, we recommend conducting a larger-scale RCT in a future outbreak. It is also important to note that testing behavior change techniques like Soapp+ during a pandemic remains challenging due to the constantly evolving context, including shifts in the pandemic trajectory, public health policies, government regulations, and medical advancements such as vaccine availability. These multifaceted and evolving factors likely contributed to the observed temporal variations in the adoption of hand hygiene behaviors, with implications for the evaluation of behavior change techniques [33,38]. For this reason, we believe that further testing of Soapp+ could extend beyond pandemic contexts and be conducted in more feasible settings, provided that the behavior change techniques specifically referring to the COVID-19 pandemic (eg, the ones targeting risk perception and outcome expectancies; Table S3 in Multimedia Appendix 1) are adapted to the specific characteristics of the new context. Upon confirmation of the current positive effect of Soapp+ on hand hygiene, this further testing beyond the pandemic context would also pave the way for a broader application of Soapp+, particularly in settings where there is a demand for hand hygiene interventions (eg, health care settings, during regular flu seasons, and to prevent the transmission of other viruses).

A methodological lesson learned regards the suitability of the MOST as a reference framework for intervention development in a pandemic context. The sequential decision-making process unique to the MOST may be too rigid for dealing with a continuously changing context. Indeed, the contextual conditions under which the intervention is developed may differ from those in which the intervention is later optimized and evaluated. Therefore, we foresee a validity risk when using the results from one phase to inform the implementation of the upcoming phase. Additionally, the pandemic context has amplified the already known challenges in completing the full MOST cycle—preparation, optimization, and evaluation—within the project timeline [40], as evidenced by the slow recruitment pace and the high dropout rates we saw in this study.

One strategy to enhance the feasibility of RCTs in future pandemics is to join forces and contribute to large-scale multicenter trials [41]. This approach was successfully adopted by observational studies investigating protective behaviors during the COVID-19 pandemic [38,42] but was less commonly used in behavior change techniques for protective behaviors, which saw fewer collaborative efforts [43]. Another strategy is represented by adaptive clinical trials [44], whose adaptive design aims to decrease the time needed to complete a trial and increase the likelihood of detecting intervention effects. Specifically, they allow for interim analysis, which is used to enhance the efficiency of the trials by adapting the original study design. Examples include response-adaptive randomization (ie, decreasing the allocation ratio to less promising arms), sample size reassessment (ie, redefining the target sample size), or seamless trials (ie, discontinuing the most unpromising arms of the trial).

### Conclusions

This study described the evaluation phase of Soapp+, a smartphone app to promote hand hygiene in the context of the COVID-19 pandemic. We leveraged digital technology and the MOST approach to address the call raised by public health experts for developing evidence-based behavior change techniques that are built to be used in a pandemic context [3]. Our findings provided tentative support for the efficacy of Soapp+, and for targeting reflective and automatic behavior change processes more broadly, in promoting hand hygiene during an ongoing pandemic. Beyond our individual results, the trial has provided important learnings for conducting rigorous trials of contextualized behavior change techniques during a pandemic, which can be further developed in the next outbreak.

## Appendix

Multimedia Appendix 1 Supplemental materials.

Multimedia Appendix 2 CONSORT-eHEALTH checklist (V 1.6.1).

## Abbreviations

CONSORT: Consolidated Standards of Reporting Trials
ITT: intention-to-treat
MOST: Multiphase Optimization Strategy
RCT: randomized controlled trial
