# Supplementary material for: Promoting Hand Hygiene During the COVID-19 Pandemic: Randomized Controlled Trial of the Optimized Soapp+ App
Source: JMIR Mhealth Uhealth. 2025 Apr 24;13:e57191. doi: 10.2196/57191 (PMC12062755; doi:10.2196/57191)
Supplement: Multimedia Appendix 1 [file mhealth_v13i1e57191_app1.docx]

**MULTIMEDIA APPENDIX 1**

Supplemental materials to the manuscript:

**Promoting Hand Hygiene During the COVID-19 Pandemic: Randomized Controlled Trial of the Optimized Soapp+ App**

Authors:

Dario Baretta, Carole Lynn Rüttimann, Melanie Alexandra Amrein, Jennifer Inauen

**Figure S1. Overview of the evaluation trial**

*
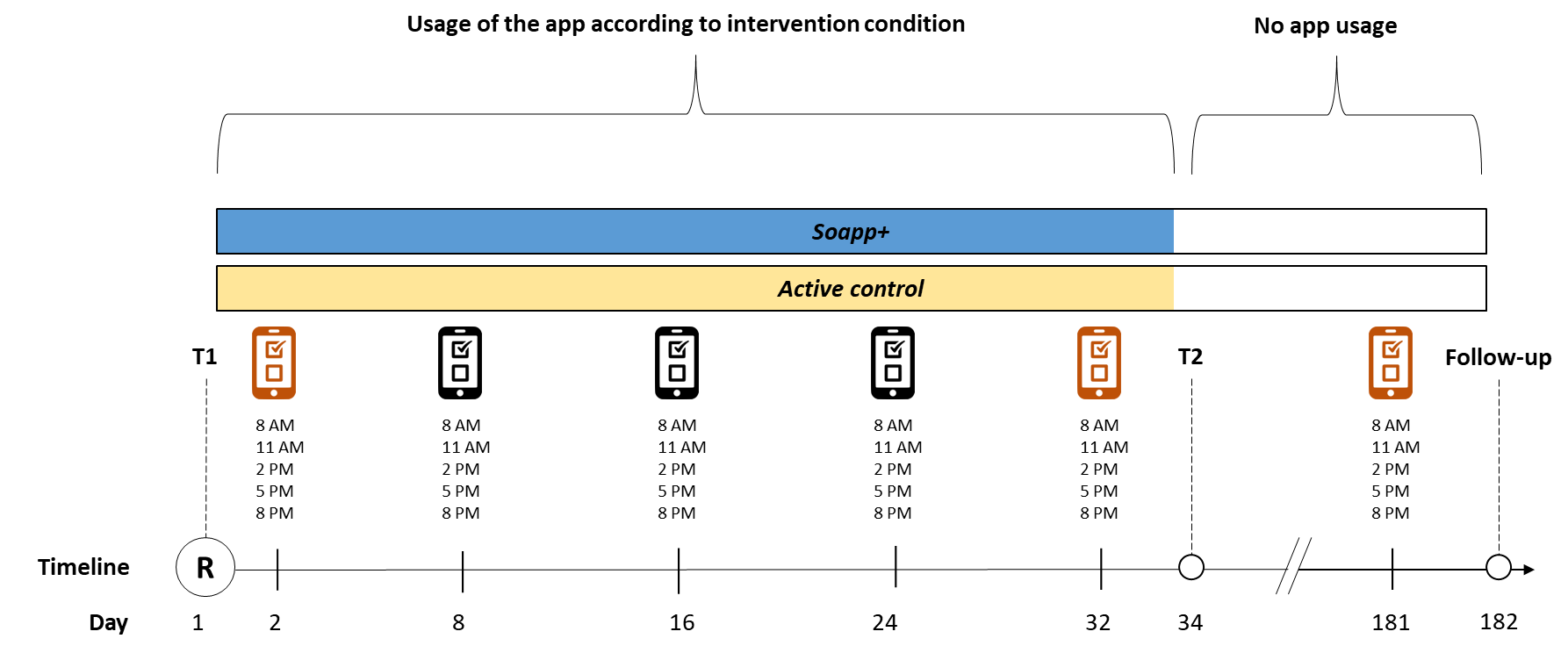
Note.* R = Randomization; Red diaries represent baseline (T1), end of the intervention (T2), and 6-month follow-up assessments for hand hygiene.

**Table S1. Socio-demographic characteristics of the Swiss permanent resident population.**

| **Variable** | **Statistic** |
| --- | --- |
| **Age (2023)** | Mean = 42.89 |
| **Sex (2022)** | |
| Male | 50% |
| Female | 50% |
| **Marital status (2023)** | |
| Single | 46.1% |
| Married | 40.3% |
| Widowed | 4.5% |
| Divorced | 8.9% |
| In a registered partnership | 0.1% |
| **Education attainment (2023) - permanent resident population aged 25 to 64 years** | |
| Compulsory education | 13.7% |
| Upper secondary level | 40.2% |
| *Vocational education and training* | *33.4%* |
| *General education* | *6.8%* |
| Tertiary level | 46.0% |
| *Vocational education and training* | *15.3%* |
| *General education* | *30.8%* |
| 90.7% of young adults up to the age of 25 will have obtained an upper secondary qualification. | |
| **Employment status (2023) – permanent resident population aged 15 and over** | |
| Self-employed and employees | 62.1% |
| Apprentices | 2.8% |
| Unemployed | 2.7% |
| People in education/training | 3.9% |
| Housewives/house husbands | 2.1% |
| Retired | 22.9% |
| Other economically inactive persons | 3.4% |
| **Gross monthly wage (2022)** | Median = 6’788 CHF |

*Note.* Information sourced from the Swiss Federal Statistical Office ([www.bfs.admin.ch](http://www.bfs.admin.ch)).

**Measures**

1. **Hand hygiene**

**Table S2. List of all key times when to perform correct hand hygiene according to the Swiss Federal Office of Public Health**

| **Item** | **Key times** | **Type** |
| --- | --- | --- |
| 1 | Before preparing the meal or before sitting down at the table | General |
| 2 | Before eating or before feeding the children | General |
| 3 | After blowing your nose, sneezing, or coughing | General |
| 4 | Every time you come home | General |
| 5 | After using public transport | General |
| 6 | After visiting sick people or after close contact with material from sick people or with their personal effects | General |
| 7 | Before inserting and removing the contact lenses | General |
| 8 | After taking off the mask | COVID-19 specific |
| 9 | After going to the toilet or accompanying a child to the toilet (including after changing diapers) | General |
| 10 | After handling waste | General |
| 11 | If you have dirty hands or if they are visibly dirty | General |
| 12 | After visiting public places | COVID-19 specific |
| 13 | After touching surfaces outside the home or money | COVID-19 specific |
| *Note.* Type = ‘General’ indicates key times that are recommended in general and before the COVID-19 pandemic occurred. | | |

1. **Self-reported flu-like infection symptoms** (assessed at T1, T2, and follow-up)

Item: *In the last four weeks, did you have flu-like infection symptoms?*

Answer options (yes / no)

1. **Self-reported statement of the occurrences of Covid-19** (assessed at T1, T2, and follow-up)

Item: *Have you had a Corona test in the last four weeks?*

Answer options (no / yes, and the result was negative (not infected) / yes, and the result was positive (infected))

**Intervention content**

**Table S3. Overview of the intervention content and targeted behavioral predictors.**

| **Condition** | **Module** | **TDF domain^a^** | **Behavioral predictor** | **Behavior change technique** | **Mode of delivery** | **Implementation** |
| --- | --- | --- | --- | --- | --- | --- |
| Intervention  Active control | Basic | Knowledge | Knowledge | 5.1 Information about health consequences | Video | Videotext: *Correct hand hygiene, i.e. correctly washing and disinfecting hands, has been omnipresent since last year at the latest. As part of the coronavirus, the Federal Office of Public Health (BAG) decided early last year that everyone in Switzerland should ensure regular hand hygiene. Regular and correct hand hygiene is an important factor in the transmission of germs, viruses or bacteria. You can protect yourself not only against the coronavirus, but also against other communicable diseases, such as seasonal flu. On the one hand, it is important that you properly disinfect and wash your hands. However, it is just as important to perform these behaviors at the right moment.* |
| Intervention  Active control | Basic | Skills | Skills | 4.1 Instruction on how to perform behavior | Video Text | The video shows how to wash hands correctly with all main steps by the FOPH. The important steps that are presented are written in the video are: *- Wet your hands under running water. - Soap your hands, if possible with liquid soap. - rub your hands until it foams, These steps are important for washing and disinfecting your hands: 1. Rub back of hand 2. Rub between your fingers 3. Rub under your fingernails 4. Rub wrists. - Rinse your hands well under running water. - Dry hands with a clean towel, if possible with a disposable paper towel or a single-use cloth towel roll.* |
| Intervention  Active control | Basic | Skills | Skills | 4.1 Instruction on how to perform behavior | Text | *Now it is also important that you wash or disinfect your hands correctly at the right moment. Here you will find a list from the FOPH with all the key times in everyday life when we should wash or disinfect our hands correctly: 1. Before preparing the meal or before sitting down at the table 2. Before eating or before feeding the children 3. after blowing your nose, sneezing or coughing 4. every time you come home 5. after using public transport 6. After visiting sick people or after close contact with material from sick people or with theirs personal effects 7. Before inserting and removing the contact lenses 8. after taking off the mask 9. after going to the toilet or accompanying a child to the toilet (including after changing diapers) 10. after handling waste 11. if you have dirty hands or if they are visibly dirty 12. After visiting public places 13. after touching surfaces outside the home or money* |
| Intervention  Active control | Basic | Goals | Intention | 1.1 Goal setting (behavior) | Text | *We ask you to read again the aim of the app soapp: Correct hand hygiene at key times.* |
| Intervention | Motivation | Beliefs about consequences | Risk perception | 5.1 Information about health consequences | Text | *As you know, the spread of many communicable diseases such as the flu or the new coronavirus can be prevented with proper hand hygiene at the key moments. Bacteria can get on your hands as soon as you touch objects or surfaces that have come into contact with someone else or with a contaminated object. Here are some facts about it: • A large source of pathogenic germs and bacteria are feces from humans or animals. If someone does not wash their hands well after going to the toilet, they are further distributed. • Contamination causes these germs and bacteria to get onto the banisters, tables, doorknobs, toys or our food. You don't see the germs and yet they are everywhere! • With proper hand washing, you can easily remove these bacteria. Unfortunately, every fifth person does not wash their hands after going to the toilet and only about a third use soap.* |
| Intervention | Motivation | Beliefs about consequences | Attitude | 5.2 Salience of consequences | Video | *Not washing your hands is gross! Check out this video:* Video showing transmission after using the bathroom  *As you can see from this example, not washing your hands is really gross.* |
| Intervention | Motivation | Goals | Intention | 1.1 Goal setting (behavior) | Text | *So remember your goal: Correct hand hygiene at key times* |
| Intervention | Motivation | Beliefs about consequences | Outcome expectancies | 9.2 Pros and cons | Text | *Today you should think about the advantages and disadvantages (pros and cons) for and against correct hand hygiene at key times. This exercise will help motivate you to practice proper hand hygiene at key times. What are the benefits of washing or disinfecting your hands correctly and at key times? [Text field] What are the downsides of washing or disinfecting your hands correctly and at key times? [Text field]* *Here you can see a graph that lists all your advantages on the one hand and all your disadvantages on the other.* [more advantages noted]: *Your weighing pan shows more advantages that speaks in favor of washing your hands correctly and at key times. So go ahead [selected call name], stay tuned!* [more disadvantages noted*]: Your weighing pan is still a bit crooked and it shows fewer advantages than disadvantages for correct hand hygiene at key times*.  Participants have the opportunity to think about it for one day.  *Did you think of any more advantages? Enter here again advantages for correct hand hygiene at key times: [Open Text field]* If still not more advantages than disadvantages are noted: *Your weighing pan is still a bit crooked and it shows fewer advantages than disadvantages for correct hand hygiene at key times.*  *Take a look at an example here where the advantages of correct hand hygiene at key times outweigh the disadvantages.*  *Possible pro entries:*  *- I protect myself from infectious diseases.*  *- I protect others from infectious diseases.*  *- A little effort on my part contributes to the prevention of infectious diseases.*  *- In the context of the current Covid-19 pandemic, I can help to interrupt the chains of transmission.*  *- I can be a role model for people around me and thus make a greater impact.*  *- I feel clean and comfortable after proper hand hygiene*  *Possible contra entries:*  *- Changing habits takes time and attention*  *- Hands are getting dry* |
| Intervention | Motivation | Beliefs about capabilities | Self-efficacy | 1.2 Problem solving | Text Video | *Changing behavior is never easy. We would like to support you in the event of difficulties. You now have the option of noting any difficulties that arise in the “Notepad” main menu. Write them down in as much detail as possible. Even if further difficulties arise in the course of the next 2 days, it is best to write them down in a notepad. Then we tackle the difficulties together.* Problem solving module will only start if participants have reported some problems (notepad). If participants haven't already reported a problem, they will first be asked if they have encountered any problem at all: *Have you observed any difficulties in the implementation in the last week? [(Yes)] Briefly describe here the difficulty you encountered. [(No)] You are on the right track, great! Keep it up!* If participants did already report some problems: *You described the following in the notepad: [contents]. Let's find solutions together!*  *To solve a problem we are going to go through the following 4 steps: 1. Describe the problem / 2. Think up different solutions / 3. Decide on a solution / 4. Implement and evaluate the solution.*  *The following video explains the 4 steps to you.* Participants can watch a video, in which all steps are explained in detail.  *Now let's go through the steps together. Here are your entries from the notepad.*  *Now decide on a difficulty that you have described and go through all 4 steps. You can write down your solution steps.*  *1. Describe the problem [Empty space to tip in]*  *2. Think up different solutions [Empty space to tip in]*  *3. Decide on a solution [Empty space to tip in]*  *4. Implement and evaluate the solution [Empty space to tip in]*  *Great, now you can try this solution: Try it the next time the problem occurs again. We ask you in two days to complete the last step «Evaluate the solution approach». Well good luck with your new solution!*  *Two days ago you defined the following solution for the difficulty you described: Were you able to implement your chosen solution? How satisfied are you with the result?* If participants are not happy with their solution: *Sometimes it doesn't work the first time, that's completely normal. Find a new solution! Maybe another way will* *work?* If participants are happy with the solution: *Great, your first solution worked great! You can repeat the whole exercise at any time in the "Solution steps" menu to tackle another problem. We wish you continued success!* People can continue with this task for all their difficulties written in their notepad. |
| Intervention | Motivation | Beliefs about capabilities | Self-efficacy | 15.1 Verbal persuasion about capabilities | Text | Participants receive push-notifications:  *- It's been almost a week now. Try to keep going. Even if it is not always easy, you can do it!* |
| Intervention | Motivation | Beliefs about consequences | Intention | 5.2 Salience of consequences | Text | Participants receive push-notifications:  *- If everyone showed correct hand hygiene, diseases could never spread so quickly. Who would want to be in bed with a fever!*  *- Small actions can make a big difference! Stay tuned and improve your hand hygiene behavior! - Brief reminder: The great thing about correct hand hygiene is that you not only protect yourself, but also others!* |
| Intervention | Motivation | Beliefs about capabilities | Self-efficacy | 15.3 Focus on past success | Text | *Hello, since you installed soapp and focused more on your hand hygiene, you have certainly experienced more successful days or days when it was more difficult despite new solutions. Try to focus during the next week, when you can wash or disinfect your hands correctly in the key situations.* Reminder: *Hello [selected call name], remember to remember your successes! Has it worked well today to wash or disinfect your hands correctly in key situations? Excellent! Keep it up!* |
| Intervention | Motivation | Reinforcement | Intention | 10.9 Self-reward | Text | *As you may have noticed before, changing behavior can be very difficult. Rewarding yourself can help you stick with your goal. A reward should do you good and ideally take place as close as possible after successfully washing or disinfecting your hands at a key point in time. For example: If I always wash my hands correctly with soap when I come home tomorrow, then I take a relaxing bath as a reward in the evening.*  Examples:  *- Meet a friend*  *- Take some time out today to do whatever you want*  *- Listen to your favorite music*  *- Take a walk*  *- Prepare your favorite tea*  *- Buy a new music album*  *- Buy yourself a new piece of clothing*  *- Buy yourself flowers*  *- Buy a fragrant body lotion*  *- Do something that you enjoy* Participants create action plans for their reward: *If (your goal for tomorrow) [Empty space to tip in goal]*  *Then (your reward) [Empty space to tip in reward]* |
| Intervention | Habit | Knowledge | Knowledge | 4.2. Information about antecedents | Video | Videotext: *Much of what we do in our everyday life is determined by a habit. There are very practical reasons for this: If behavior is controlled by a habit, we do not have to think about what and how we are doing something - we then act automatically and unconsciously. But what exactly is a habit? A habit is a trigger for behavior. This is in turn triggered by a notice. For example, you may have noticed that when you get into a vehicle, you immediately buckle your seat belt without thinking. In this case, you should get into the vehicle as a hint. We now want to transfer this mechanism to hand hygiene with the help of this module. We want to help you make correct hand hygiene a key part of making a habit. How does a habit develop? A behavior has to be repeated until an association between the triggering cue and the behavior arises in our brain. Research has shown that action plans help build habits. An action plan defines when and how you show behavior.* |
| Intervention | Habit | Memory, attention, and decision processes | Action control | 2.3 Self-monitoring of behavior | Video  Text | *The next step is to identify key situations in which you are not yet washing or disinfecting your hands correctly. This app offers you a diary for this. For the next two days, when you open the app, the diary will automatically open so that you can quickly take a note. Now observe yourself in your everyday life for the next two days. Make a note of the specific situations in which you should wash or disinfect your hands, but not yet do this on a regular basis. Then open the app and write this specific situation down in your notepad.*  REMINDER: *You have not yet recorded anything in your diary. Please define a situation in which you should wash or disinfect your hands, so that you can continue with this task.* |
| Intervention | Habit | Goals | Action planning | 1.4 Action planning  7.1. Prompts/cues | Text | *You will now create an initial plan of action. In the action plan, you now write down exactly what you intend to do in specific situations. The goal is thus a chain of behavior. Let's look at this in an example: Laura notes that when she comes home she (1) takes off your shoes, then (2) hangs up the jacket then (3) put the bag in the living room and last (4) washes her hands. Laura writes down the following plan of action: "When I get home, I take off my shoes, hang up my jacket and then wash my hands" Create suitable action plans for you: You can formulate action plans for each of your specific situations from the notepad, like Laura: [Empty bracket where participants can enter their action plan] -> [Empty bracket where participants can enter their action plan] -> Wash / disinfect hands During the next three days, you can also change your action plans there or add new ones. Not every action plan works right away. Just take a look at how it works over the next three days. We'll remind you of this every now and then.* PUSH NOTIFICATION *Hey, how are your plans of action going? As a reminder, you can change them or add new ones at any time.* |
| Intervention | Habit | Skills | Habit strength | 8.3 Habit formation 8.1 Behavioral practice/ rehearsal | Text | *Soapp would be happy to help you to perform hand hygiene by which you can receive message at a certain time. Would you like a reminder? Yes/No Only select this option if you want to wash or disinfect your hands at around the same time. For example, normally you cook your dinner at 6pm every day. If you select 6pm as your requested time, soapp will send you a reminder every day in the next at this time.* I want a reminder at [people can indicate time] Option: I'd rather not receive a message. REMINDER at the selected time points: *[selected call name], don't forget to wash or disinfect your hands right away!* |
| Intervention | Habit | Skills | Habit strength | 8.3 Habit formation 8.1 Behavioral practice/ rehearsal | Text | *Now you can change your action plan the last time. Do you with you change them again?* Yes / no.  *So [Username], let’s go! Try to perform your fixed action plans as much as possible in the next couple of days. We will ask you sometimes how it goes.* |
| Intervention | Habit | Goals | Habit strength | 8.3 Habit formation  8.1 Behavioral practice/ rehearsal | Text | Participants receive push-notification during the module  PUSH NOTIFICATION *Hey, how are your plans of action going? We wish you great success to perform correct hand hygiene at key times*. |
| Intervention | Habit | Behavioral regulation | Habit strength | 7.1 Prompts/cues (physical cue) | Text Pictures | *Set physical cues in your environment. This can help you that you can develop a habit. You can set yourself, for example, a post-it note or pictures, etc. You can determine the information on your post-it or the picture completely freely and it does not have to have anything to do with washing your hands. Take a look at these funny pictures as input. Find funny pictures and place them where they remind you of your plan of action (e.g. in the cloakroom, in the kitchen, etc.).* |
| ^a^TDF: theoretical domains framework | | | | | | |

Figure S2. Intervention timeline

| **Intervention day** | **1** | **2** | **3** | **4** | **5** | **6** | **7** | **8** | **9** | **10** | **11** | **12** | **13** | **14** | **15** | **16** | **17** | **18** | **19** | **20** | **21** | **22** | **23** | **24** | **25** | **26** | **27** | **28** | **29** | **30** | **31** | **32** | **33** | **34** |
| --- | --- | --- | --- | --- | --- | --- | --- | --- | --- | --- | --- | --- | --- | --- | --- | --- | --- | --- | --- | --- | --- | --- | --- | --- | --- | --- | --- | --- | --- | --- | --- | --- | --- | --- |
| Panel assessment (T1-T2) ^1,2^ |  |  |  |  |  |  |  |  |  |  |  |  |  |  |  |  |  |  |  |  |  |  |  |  |  |  |  |  |  |  |  |  |  |  |
| Hand hygiene diaries^1,2^ |  |  |  |  |  |  |  |  |  |  |  |  |  |  |  |  |  |  |  |  |  |  |  |  |  |  |  |  |  |  |  |  |  |  |
| Basic module^1,2^ |  |  |  |  |  |  |  |  |  |  |  |  |  |  |  |  |  |  |  |  |  |  |  |  |  |  |  |  |  |  |  |  |  |  |
| Tasks | | | | | | | | | | | | | | | | | | | | | | | | | | | | | | | | | | |
| Quizzes^2^ |  |  |  |  |  |  |  |  |  |  |  |  |  |  |  |  |  |  |  |  |  |  |  |  |  |  |  |  |  |  |  |  |  |  |
| Action planning^1^ |  |  |  |  |  |  |  |  |  |  |  |  |  |  |  |  |  |  |  |  |  |  |  |  |  |  |  |  |  |  |  |  |  |  |
| Habit formation^1^ |  |  |  |  |  |  |  |  |  |  |  |  |  |  |  |  |  |  |  |  |  |  |  |  |  |  |  |  |  |  |  |  |  |  |
| Setting reminder*^1^ |  |  |  |  |  |  |  |  |  |  |  |  |  |  |  |  |  |  |  |  |  |  |  |  |  |  |  |  |  |  |  |  |  |  |
| Physical cue*^1^ |  |  |  |  |  |  |  |  |  |  |  |  |  |  |  |  |  |  |  |  |  |  |  |  |  |  |  |  |  |  |  |  |  |  |
| Motivational video^1^ |  |  |  |  |  |  |  |  |  |  |  |  |  |  |  |  |  |  |  |  |  |  |  |  |  |  |  |  |  |  |  |  |  |  |
| Pros and Cons^1^ |  |  |  |  |  |  |  |  |  |  |  |  |  |  |  |  |  |  |  |  |  |  |  |  |  |  |  |  |  |  |  |  |  |  |
| Problem solving^1^ |  |  |  |  |  |  |  |  |  |  |  |  |  |  |  |  |  |  |  |  |  |  |  |  |  |  |  |  |  |  |  |  |  |  |
| Self-incentive^1^ |  |  |  |  |  |  |  |  |  |  |  |  |  |  |  |  |  |  |  |  |  |  |  |  |  |  |  |  |  |  |  |  |  |  |
| Motivational message^1^ |  |  |  |  |  |  |  |  |  |  |  |  |  |  |  |  |  |  |  |  |  |  |  |  |  |  |  |  |  |  |  |  |  |  |

*Note*. ^1^ Content delivered to the intervention group; ^2^ Content delivered to the active control group. Color meaning: *i*) Green squares represent the assessment points for panel surveys and hand hygiene diaries; *ii*) Yellow squares represent the content of the basic module which was always accessible throughout the intervention phase to both groups. Bright yellow squares indicate the days when the fun fact notifications were delivered. *iii*)Orange squares indicate the days when the quizzes were delivered to the active control group. *iv*) Blue squares indicate the days when tasks from the habit or motivation module were active for the intervention group. * The tasks were optionally available to the participants.

**Sensitivity analysis**

**Table S4. Differences in changes in hand hygiene and behavioral determinants between intervention group and active control group with the Student *t* test.**

| **Variable** | **Change** | **N** | **Overall***^1^* | **Active control group***^1^* | **Intervention group***^1^* | **Statistic** | **p-value** | **Cohen’s *d*** |
| --- | --- | --- | --- | --- | --- | --- | --- | --- |
| ***Target behavior*** |  |  |  |  |  |  |  |  |
| Hand hygiene | T1-T2 | 143 | 0.09 (0.72) | -0.02 (0.76) | 0.18 (0.67) | -1.654 | 0.10 | 0.28 |
| Hand hygiene | T1-Follow-up | 143 | -0.08 (0.72) | -0.21 (0.80) | 0.02 (0.62) | -1.848 | 0.07 | 0.31 |
| ***Behavioral determinants*** |  |  |  |  |  |  |  |  |
| Intention | T1-T2 | 193 | 0.05 (0.66) | 0.06 (0.59) | 0.03 (0.84) | 0.334 | 0.74 | 0.05 |
| Intention | T1-Follow-up | 193 | -0.15 (0.76) | -0.13 (0.71) | -0.16 (0.67) | 0.365 | 0.72 | 0.05 |
| Action planning | T1-T2 | 193 | 0.16 (0.90) | 0.08 (0.77) | 0.25 (1.01) | -1.352 | 0.18 | 0.20 |
| Action planning | T1-Follow-up | 193 | 0.00 (1.03) | 0.01 (0.96) | -0.02 (1.11) | 0.232 | 0.82 | 0.03 |
| Coping planning | T1-T2 | 193 | 0.33 (0.98) | 0.19 (0.85) | 0.46 (1.08) | -1.884 | 0.06 | 0.27 |
| Coping planning | T1-Follow-up | 193 | 0.19 (1.07) | 0.17 (1.08) | 0.22 (1.06) | -0.323 | 0.75 | 0.05 |
| Habit strength | T1-T2 | 187 | 0.08 (0.88) | 0.11 (0.79) | 0.05 (0.97) | 0.421 | 0.67 | 0.06 |
| Habit strength | T1-Follow-up | 187 | 0.00 (1.03) | 0.04 (1.04) | -0.05 (1.03) | 0.567 | 0.57 | 0.08 |
| Action control | T1-T2 | 152 | 0.35 (0.94) | 0.31 (0.93) | 0.39 (0.95) | -0.475 | 0.64 | 0.08 |
| Action control | T1-Follow-up | 152 | 0.07 (1.00) | 0.09 (1.12) | 0.05 (0.86) | 0.203 | 0.84 | 0.03 |
| Self-efficacy | T1-T2 | 193 | 0.10 (0.52) | 0.08 (0.55) | 0.11 (0.49) | -0.331 | 0.74 | 0.05 |
| Self-efficacy | T1-Follow-up | 193 | -0.04 (0.67) | -0.02 (0.74) | -0.06 (0.59) | 0.455 | 0.65 | 0.07 |
| Attitudes | T1-T2 | 188 | -0.05 (0.66) | -0.06 (0.75) | -0.05 (0.55) | -0.099 | 0.92 | 0.01 |
| Attitudes | T1-Follow-up | 188 | -0.18 (0.84) | -0.19 (0.94) | -0.17 (0.74) | -0.208 | 0.87 | 0.03 |
| Outcome expectancies | T1-T2 | 193 | 0.04 (0.45) | 0.09 (0.38) | 0.00 (0.51) | 1.445 | 0.15 | 0.21 |
| Outcome expectancies | T1-Follow-up | 193 | -0.04 (0.56) | 0.01 (0.55) | -0.09 (0.58) | 1.240 | 0.22 | 0.18 |
| Risk Perception | T1-T2 | 193 | -0.04 (0.63) | -0.08 (0.50) | 0.00 (0.75) | -0.823 | 0.41 | 0.12 |
| Risk Perception | T1-Follow-up | 193 | -0.09 (0.73) | -0.19 (0.63) | 0.00 (0.82) | -1.806 | 0.07 | 0.26 |

Note. *^1^*Mean (SD)

**Table S5. Paired Wilcoxon tests of behavioral determinants over time**

| **Variable** | **Comparison** | **N** | **W-Statistic** | **p-value** |
| --- | --- | --- | --- | --- |
| Intention | T1-T2 | 193 | 563 | 0.13 |
|  | T1-Follow-up | 193 | 1542 | 0.006 |
| Action planning | T1-T2 | 193 | 1338 | 0.006 |
|  | T1-Follow-up | 193 | 3114 | 0.88 |
| Coping planning | T1-T2 | 193 | 1474 | <0.001 |
|  | T1-Follow-up | 193 | 3322 | 0.02 |
| Habit strength | T1-T2 | 187 | 2254 | 0.28 |
|  | T1-Follow-up | 187 | 3995 | 0.89 |
| Action control | T1-T2 | 152 | 611 | <0.001 |
|  | T1-Follow-up | 152 | 1758 | 0.41 |
| Self-efficacy | T1-T2 | 193 | 2115 | 0.01 |
|  | T1-Follow-up | 193 | 4742 | 0.74 |
| Attitudes | T1-T2 | 188 | 2067 | 0.92 |
|  | T1-Follow-up | 188 | 4650 | 0.01 |
| Outcome expectancies | T1-T2 | 193 | 2282 | 0.15 |
|  | T1-Follow-up | 193 | 4530 | 0.34 |
| Risk perception | T1-T2 | 193 | 2721 | 0.22 |
|  | T1-Follow-up | 193 | 4759 | 0.07 |

**Table S6. Sensitivity analysis without missing values imputation in hand hygiene and targeted behavioral determinants at each assessment point and Shapiro-Wilk normality test (equivalent to Table 3 in the main paper).**

|  |  |  |  | | | **Shapiro-Wilk normality test** | |
| --- | --- | --- | --- | --- | --- | --- | --- |
| **Variable** | **Assessment point** | **N** | **Overall***^1^* | **Active control group***^1^* | **Intervention group***^1^* | **Statistic** | **p-value** |
| ***Target behavior*** | | | | | | | |
| Hand hygiene | T1 | 143 | 3.25 (2.63, 3.60) | 3.23 (2.56, 3.57) | 3.25 (2.71, 3.61) | 0.897 | <0.001 |
|  | T2 | 90 | 3.53 (2.69, 3.91) | 3.24 (2.59, 3.85) | 3.63 (3.16, 4.00) | 0.834 | <0.001 |
|  | Follow-up | 105 | 3.25 (2.50, 3.69) | 3.17 (2.48, 3.60) | 3.27 (2.79, 3.77) | 0.882 | <0.001 |
| ***Behavioral determinants*** | | | | | | | |
| Intention | T1 | 193 | 5.00 (5.00, 6.00) | 5.00 (5.00, 5.25) | 5.00 (5.00, 6.00) | 0.823 | <0.001 |
|  | T2 | 117 | 5.00 (5.00, 6.00) | 5.00 (5.00, 6.00) | 5.00 (5.00, 5.00) | 0.783 | <0.001 |
|  | Follow-up | 136 | 5.00 (4.00, 5.00) | 5.00 (4.00, 5.00) | 5.00 (4.00, 5.00) | 0.848 | <0.001 |
| Action planning | T1 | 193 | 4.33 (4.00, 5.00) | 4.33 (3.67, 5.00) | 4.67 (4.00, 5.00) | 0.922 | <0.001 |
|  | T2 | 117 | 5.00 (4.00, 5.33) | 5.00 (4.00, 5.33) | 5.00 (4.00, 5.00) | 0.885 | <0.001 |
|  | Follow-up | 136 | 4.33 (3.67, 5.00) | 4.33 (3.67, 5.00) | 4.50 (3.67, 5.00) | 0.923 | <0.001 |
| Coping planning | T1 | 193 | 3.75 (2.75, 4.25) | 3.50 (2.75, 4.25) | 3.75 (2.75, 4.50) | 0.974 | 0.001 |
|  | T2 | 117 | 4.25 (3.50, 4.75) | 4.00 (3.25, 4.75) | 4.50 (3.94, 5.00) | 0.957 | 0.001 |
|  | Follow-up | 136 | 3.75 (2.75, 4.75) | 3.75 (2.50, 4.50) | 3.88 (3.00, 5.00) | 0.965 | 0.001 |
| Habit strength | T1 | 187 | 5.25 (4.00, 6.25) | 5.00 (3.50, 6.00) | 5.75 (4.19, 6.25) | 0.933 | <0.001 |
|  | T2 | 116 | 5.25 (4.25, 6.00) | 5.50 (4.25, 6.00) | 5.25 (4.50, 5.88) | 0.955 | 0.001 |
|  | Follow-up | 136 | 5.25 (4.25, 6.00) | 5.25 (3.81, 6.19) | 5.00 (4.25, 6.00) | 0.930 | <0.001 |
| Action control | T1 | 152 | 4.00 (3.33, 5.00) | 4.00 (3.58, 5.00) | 4.00 (3.33, 5.00) | 0.947 | <0.001 |
|  | T2 | 113 | 4.67 (4.00, 5.33) | 4.33 (4.00, 5.00) | 5.00 (4.33, 5.33) | 0.954 | 0.001 |
|  | Follow-up | 119 | 4.33 (3.33, 5.00) | 4.50 (3.33, 5.00) | 4.33 (3.42, 5.00) | 0.957 | 0.001 |
| Self-efficacy | T1 | 193 | 4.25 (3.63, 4.88) | 4.13 (3.50, 4.75) | 4.38 (3.63, 5.00) | 0.986 | 0.049 |
|  | T2 | 116 | 4.50 (3.84, 5.00) | 4.38 (3.88, 5.13) | 4.50 (3.81, 5.00) | 0.969 | 0.008 |
|  | Follow-up | 136 | 4.25 (3.47, 4.88) | 4.25 (3.41, 5.00) | 4.19 (3.63, 4.75) | 0.983 | 0.09 |
| Attitudes | T1 | 188 | 5.17 (4.67, 5.62) | 5.17 (4.67, 5.50) | 5.17 (4.67, 5.67) | 0.932 | <0.001 |
|  | T2 | 116 | 5.08 (4.50, 5.50) | 5.17 (4.50, 5.50) | 5.00 (4.42, 5.50) | 0.877 | <0.001 |
|  | Follow-up | 136 | 5.00 (4.33, 5.50) | 5.00 (4.33, 5.33) | 5.00 (4.33, 5.67) | 0.900 | <0.001 |
| Outcome expectancies | T1 | 193 | 4.63 (4.25, 5.13) | 4.63 (4.25, 5.00) | 4.75 (4.25, 5.13) | 0.979 | 0.005 |
|  | T2 | 117 | 4.75 (4.25, 5.13) | 4.75 (4.25, 5.25) | 4.75 (4.22, 5.13) | 0.979 | 0.07 |
|  | Follow-up | 136 | 4.63 (4.13, 5.03) | 4.63 (4.13, 5.00) | 4.63 (4.16, 5.09) | 0.987 | 0.21 |
| Risk perception | T1 | 193 | 4.60 (4.00, 5.00) | 4.60 (4.00, 5.00) | 4.60 (3.80, 5.00) | 0.942 | <0.001 |
|  | T2 | 117 | 4.40 (3.80, 5.00) | 4.60 (3.80, 5.00) | 4.40 (3.90, 5.00) | 0.968 | 0.007 |
|  | Follow-up | 136 | 4.40 (3.60, 5.00) | 4.30 (3.60, 5.00) | 4.60 (4.00, 5.00) | 0.963 | 0.001 |

Note. *^1^*Median (IQR)

**Table S7. Sensitivity analysis without missing values imputation for differences in changes in hand hygiene and behavioral determinants between intervention group and active control group (equivalent to Table 4 in the main paper).**

| **Variable** | **Change** | **N** | **Overall***^1^* | **Active control group***^1^* | **Intervention group***^1^* | **W-Statistic** | **p-value** | **Effect size *r****^2^* |
| --- | --- | --- | --- | --- | --- | --- | --- | --- |
| ***Target behavior*** | | | | | | | | |
| Hand hygiene | T1-T2 | 89 | 0.15 (-0.17, 0.53) | -0.01 (-0.50, 0.45) | 0.32 (0.00, 0.58) | 738 | 0.04 | 0.22 |
|  | T1-Follow-up | 95 | 0.00 (-0.47, 0.26) | -0.09 (-0.68, 0.19) | 0.07 (-0.24, 0.32) | 1061 | 0.04 | 0.20 |
| ***Behavioral determinants*** | | | | | | | | |
| Intention | T1-T2 | 117 | 0.00 (0.00, 1.00) | 0.00 (0.00, 0.00) | 0.00 (0.00, 1.00) | 1613 | 0.64 | 0.04 |
|  | T1-Follow-up | 136 | 0.00 (-1.00, 0.00) | 0.00 (0.00, 0.00) | 0.00 (-1.00, 0.00) | 2462 | 0.47 | 0.06 |
| Action planning | T1-T2 | 117 | 0.33 (0.00, 0.67) | 0.33 (-0.33, 0.67) | 0.33 (0.00, 1.33) | 1409 | 0.12 | 0.14 |
|  | T1-Follow-up | 136 | 0.00 (-0.67, 0.67) | 0.00 (-0.58, 0.33) | 0.00 (-0.67, 0.67) | 2325 | 0.95 | 0.01 |
| Coping planning | T1-T2 | 117 | 0.50 (-0.25, 1.50) | 0.25 (-0.50, 1.00) | 0.88 (0.00, 1.81) | 1151 | 0.003 | 0.27 |
|  | T1-Follow-up | 136 | 0.25 (-0.50, 1.00) | 0.00 (-0.50, 0.75) | 0.38 (-0.50, 1.00) | 2131 | 0.43 | 0.07 |
| Habit strength | T1-T2 | 115 | 0.00 (-0.50, 0.75) | 0.00 (-0.50, 0.75) | 0.00 (-0.75, 0.75) | 1717 | 0.60 | 0.05 |
|  | T1-Follow-up | 132 | 0.00 (-0.75, 0.75) | 0.00 (-0.75, 0.75) | 0.00 (-0.75, 0.63) | 2205 | 0.89 | 0.01 |
| Action control | T1-T2 | 91 | 0.33 (0.00, 1.00) | 0.33 (0.00, 1.00) | 0.33 (0.00, 1.00) | 931 | 0.50 | 0.07 |
|  | T1-Follow-up | 98 | 0.00 (-0.63, 0.67) | 0.00 (-0.58, 0.67) | 0.00 (-0.50, 0.50) | 1219 | 0.89 | 0.01 |
| Self-efficacy | T1-T2 | 116 | 0.13 (-0.25, 0.50) | 0.13 (-0.25, 0.38) | 0.25 (-0.19, 0.69) | 1552 | 0.55 | 0.06 |
|  | T1-Follow-up | 136 | 0.00 (-0.50, 0.50) | 0.00 (-0.50, 0.47) | 0.00 (-0.50, 0.47) | 2422 | 0.63 | 0.04 |
| Attitudes | T1-T2 | 115 | 0.00 (-0.25, 0.33) | 0.00 (-0.17, 0.33) | 0.00 (-0.33, 0.33) | 1726 | 0.57 | 0.05 |
|  | T1-Follow-up | 132 | 0.00 (-0.33, 0.17) | 0.00 (-0.33, 0.17) | -0.17 (-0.42, 0.17) | 2383 | 0.34 | 0.08 |
| Outcome expectancies | T1-T2 | 117 | 0.13 (-0.25, 0.38) | 0.13 (-0.13, 0.38) | 0.06 (-0.50, 0.41) | 1892 | 0.27 | 0.10 |
|  | T1-Follow-up | 136 | 0.00 (-0.50, 0.38) | 0.00 (-0.38, 0.25) | -0.13 (-0.63, 0.38) | 2545 | 0.31 | 0.09 |
| Risk Perception | T1-T2 | 117 | 0.00 (-0.60, 0.40) | 0.00 (-0.40, 0.20) | 0.00 (-0.60, 0.40) | 1575 | 0.53 | 0.06 |
|  | T1-Follow-up | 136 | -0.10 (-0.65, 0.40) | -0.20 (-0.80, 0.20) | 0.00 (-0.60, 0.60) | 1828 | 0.04 | 0.18 |

*Note. ^1^*Median (IQR). *^2^* The following guidelines are used to interpret the effect size (r): a large effect is defined as *r* ≥ .50, a medium effect as *r* ≈ .30, and a small effect as *r* ≈ .10 (Fritz et al., 2012)

**Secondary analysis on flu-like symptoms or positivity to COVID-19**

**Table S8. Number of participants reporting flu-like symptoms or positivity to COVID-19 (i.e., positive test) at each time point.**

| **Time** | **Number of participants reporting flu like symptoms in the previous 4 weeks (%, number of respondents)** | | **Number of participants resulting positive to a COVID-19 test in the previous 4 weeks (%, number of respondents)** | |
| --- | --- | --- | --- | --- |
|  | **Intervention** | **Active control** | **Intervention** | **Active control** |
| T1 | 24 (25%, 97) | 21 (22%, 96) | 7 (7%, 97) | 6 (6%, 96) |
| T2 | 12 (22%, 55) | 20 (31%, 65) | 2 (4%, 55) | 7 (11%, 65) |
| Follow-up | 16 (22%, 72) | 23 (32%, 73) | 3 (4%, 73) | 0 (0%, 71) |

**Chi-square tests**

Pearson Chi-square test suggested that there were no group differences in:

- Self-reported flu-like symptoms at T2: *X^2^*= 0.806, df = 1, *P* = 0.369
- Self-reported flu-like symptoms at follow-up: *X^2^*= 0.950, df = 1, *P* = 0.330
- Self-reported positive testing to COVID-19 at T2: *X^2^*= 1.278, df = 1, *P* = 0.258
- Self-reported positive testing to COVID-19 at follow-up: *X^2^*= 1.455, df = 1, *P* = 0.228
