# Supplementary material for: Promoting Hand Hygiene During the COVID-19 Pandemic: Randomized Controlled Trial of the Optimized Soapp+ App
Source: JMIR Mhealth Uhealth. 2025 Apr 24;13:e57191. doi: 10.2196/57191 (PMC12062755; doi:10.2196/57191)
Supplement: Multimedia Appendix 2 [file mhealth_v13i1e57191_app2.pdf]

CONSORT-EHEALTH (V 1.6.1) - Submission/Publication Form

The CONSORT-EHEALTH checklist is intended for authors of randomized trials evaluating web-based and Internet-based applications/interventions, including mobile interventions, electronic games (incl multiplayer games), social media, certain telehealth applications, and other interactive and/or networked electronic applications. Some of the items (e.g. all subitems under item 5 - description of the intervention) may also be applicable for other study designs.

The goal of the CONSORT EHEALTH checklist and guideline is to be
a) a guide for reporting for authors of RCTs,
b) to form a basis for appraisal of an ehealth trial (in terms of validity)

CONSORT-EHEALTH items/subitems are MANDATORY reporting items for studies published in the Journal of Medical Internet Research and other journals / scientific societies endorsing the checklist.

Items numbered 1., 2., 3., 4a., 4b etc are original CONSORT or CONSORT-NPT (non-pharmacologic treatment) items.
Items with Roman numerals (i., ii, iii, iv etc.) are CONSORT-EHEALTH extensions/clarifications.

As the CONSORT-EHEALTH checklist is still considered in a formative stage, we would ask that you also RATE ON A SCALE OF 1-5 how important/useful you feel each item is FOR THE PURPOSE OF THE CHECKLIST and reporting guideline (optional).

Mandatory reporting items are marked with a red *.
In the textboxes, either copy & paste the relevant sections from your manuscript into this form - please include any quotes from your manuscript in QUOTATION MARKS,
or answer directly by providing additional information not in the manuscript, or elaborating on why the item was not relevant for this study.

YOUR ANSWERS WILL BE PUBLISHED AS A SUPPLEMENTARY FILE TO YOUR PUBLICATION IN JMIR AND ARE CONSIDERED PART OF YOUR PUBLICATION (IF ACCEPTED).
Please fill in these questions diligently. Information will not be copyedited, so please use proper spelling and grammar, use correct capitalization, and avoid abbreviations.

DO NOT FORGET TO SAVE AS PDF _AND_ CLICK THE SUBMIT BUTTON SO YOUR ANSWERS ARE IN OUR DATABASE !!!

Citation Suggestion (if you append the pdf as Appendix we suggest to cite this paper in the caption):
Eysenbach G, CONSORT-EHEALTH Group

CONSORT-EHEALTH: Improving and Standardizing Evaluation Reports of Web-based and Mobile Health Interventions
J Med Internet Res 2011; 13(4):e126
URL: http://www.jmir.org/2011/4/e126/
doi: 10.2196/jmir.1923
PMID: 22209829

Sign in to Google to save your progress. Learn more

* Indicates a required question

Your name *
First Last

Baretta Dario

Primary Affiliation (short), City, Country *
University of Toronto, Toronto, Canada

University of Bern, Bern, Switzerland

Your e-mail address *
abc@gmail.com

dario.baretta@unibe.ch

Title of your manuscript *
Provide the (draft) title of your manuscript.

Promoting hand hygiene during the COVID-19 pandemic: A randomized controlled trial of the optimized Soapp+ app

Name of your App/Software/Intervention *

If there is a short and a long/alternate name, write the short name first and add the long name in brackets.

Soapp+

Evaluated Version (if any)

e.g. "V1", "Release 2017-03-01", "Version 2.0.27913"

My answer

Language(s) *

What language is the intervention/app in? If multiple languages are available, separate by comma (e.g. "English, French")

German

URL of your Intervention Website or App

e.g. a direct link to the mobile app on app in appstore (itunes, Google Play), or URL of the website. If the intervention is a DVD or hardware, you can also link to an Amazon page.

https://apps.apple.com/us/app/soapp/id1607534056

URL of an image/screenshot (optional)

My answer

Accessibility *

Can an enduser access the intervention presently?

- ○ access is free and open
- ○ access only for special usergroups, not open
- ○ access is open to everyone, but requires payment/subscription/in-app purchases
- ◉ app/intervention no longer accessible
- ○ Other:

Primary Medical Indication/Disease/Condition *

e.g. "Stress", "Diabetes", or define the target group in brackets after the condition, e.g. "Autism (Parents of children with)", "Alzheimers (Informal Caregivers of)"

COVID-19 (Preventing spread of)

Primary Outcomes measured in trial *

comma-separated list of primary outcomes reported in the trial

Hand hygiene

Secondary/other outcomes

Are there any other outcomes the intervention is expected to affect?

Flu-like infection symptoms and occurrences of Covid-19. Additionally, psychological derminants of hand hygiene

Recommended "Dose" *

What do the instructions for users say on how often the app should be used?

- (x) Approximately Daily
- ( ) Approximately Weekly
- ( ) Approximately Monthly
- ( ) Approximately Yearly
- ( ) "as needed"
- ( ) Other:

Approx. Percentage of Users (starters) still using the app as recommended after 3 months *

- ( ) unknown / not evaluated
- ( ) 0-10%
- ( ) 11-20%
- ( ) 21-30%
- ( ) 31-40%
- ( ) 41-50%
- ( ) 51-60%
- ( ) 61-70%
- ( ) 71%-80%
- ( ) 81-90%
- ( ) 91-100%
- (x) Other: The intervention lasted less than 3 months (32 days)

Overall, was the app/intervention effective? *

- (selected) yes: all primary outcomes were significantly better in intervention group vs control
- partly: SOME primary outcomes were significantly better in intervention group vs control
- no statistically significant difference between control and intervention
- potentially harmful: control was significantly better than intervention in one or more outcomes
- inconclusive: more research is needed
- Other:

Article Preparation Status/Stage *

At which stage in your article preparation are you currently (at the time you fill in this form)

- not submitted yet - in early draft status
- not submitted yet - in late draft status, just before submission
- submitted to a journal but not reviewed yet
- (selected) submitted to a journal and after receiving initial reviewer comments
- submitted to a journal and accepted, but not published yet
- published
- Other:

Journal *

If you already know where you will submit this paper (or if it is already submitted), please provide the journal name (if it is not JMIR, provide the journal name under "other")

- ○ not submitted yet / unclear where I will submit this
- ○ Journal of Medical Internet Research (JMIR)
- ◉ JMIR mHealth and UHealth
- ○ JMIR Serious Games
- ○ JMIR Mental Health
- ○ JMIR Public Health
- ○ JMIR Formative Research
- ○ Other JMIR sister journal
- ○ Other:

Is this a full powered effectiveness trial or a pilot/feasibility trial? *

- ○ Pilot/feasibility
- ◉ Fully powered

Manuscript tracking number *

If this is a JMIR submission, please provide the manuscript tracking number under "other" (The ms tracking number can be found in the submission acknowledgement email, or when you login as author in JMIR. If the paper is already published in JMIR, then the ms tracking number is the four-digit number at the end of the DOI, to be found at the bottom of each published article in JMIR)

- ○ no ms number (yet) / not (yet) submitted to / published in JMIR
- ◉ Other: 57191

TITLE AND ABSTRACT

1a) TITLE: Identification as a randomized trial in the title

1a) Does your paper address CONSORT item 1a? *

I.e does the title contain the phrase "Randomized Controlled Trial"? (if not, explain the reason under "other")

- (selected) yes
- Other:

1a-i) Identify the mode of delivery in the title

Identify the mode of delivery. Preferably use “web-based” and/or “mobile” and/or “electronic game” in the title. Avoid ambiguous terms like “online”, “virtual”, “interactive”. Use “Internet-based” only if Intervention includes non-web-based Internet components (e.g. email), use “computer-based” or “electronic” only if offline products are used. Use “virtual” only in the context of “virtual reality” (3-D worlds). Use “online” only in the context of “online support groups”. Complement or substitute product names with broader terms for the class of products (such as “mobile” or “smart phone” instead of “iphone”), especially if the application runs on different platforms.

	1	2	3	4	5	
subitem not at all important	○	○	●	○	○	essential

Clear selection

Does your paper address subitem 1a-i? *

Copy and paste relevant sections from manuscript title (include quotes in quotation marks "like this" to indicate direct quotes from your manuscript), or elaborate on this item by providing additional information not in the ms, or briefly explain why the item is not applicable/relevant for your study

yes, the title refer to a smartphone application: "Promoting hand hygiene during the COVID-19 pandemic: A randomized controlled trial of the optimized Soapp+ app"

1a-ii) Non-web-based components or important co-interventions in title

Mention non-web-based components or important co-interventions in title, if any (e.g., “with telephone support”).

	1	2	3	4	5	
subitem not at all important	○	◉	○	○	○	essential

Clear selection

Does your paper address subitem 1a-ii?

Copy and paste relevant sections from manuscript title (include quotes in quotation marks "like this" to indicate direct quotes from your manuscript), or elaborate on this item by providing additional information not in the ms, or briefly explain why the item is not applicable/relevant for your study

Sub-item 1a-ii is not relevant in this manuscript

1a-iii) Primary condition or target group in the title

Mention primary condition or target group in the title, if any (e.g., “for children with Type I Diabetes”) Example: A Web-based and Mobile Intervention with Telephone Support for Children with Type I Diabetes: Randomized Controlled Trial

	1	2	3	4	5	
subitem not at all important	○	○	○	◉	○	essential

Clear selection

Does your paper address subitem 1a-iii? *

Copy and paste relevant sections from manuscript title (include quotes in quotation marks "like this" to indicate direct quotes from your manuscript), or elaborate on this item by providing additional information not in the ms, or briefly explain why the item is not applicable/relevant for your study

The study presented in the manuscript focuses on the general population, therefore it is not specified in the title.

1b) ABSTRACT: Structured summary of trial design, methods, results, and conclusions

NPT extension: Description of experimental treatment, comparator, care providers, centers, and blinding status.

1b-i) Key features/functionalities/components of the intervention and comparator in the METHODS section of the ABSTRACT

Mention key features/functionalities/components of the intervention and comparator in the abstract. If possible, also mention theories and principles used for designing the site. Keep in mind the needs of systematic reviewers and indexers by including important synonyms. (Note: Only report in the abstract what the main paper is reporting. If this information is missing from the main body of text, consider adding it)

	1	2	3	4	5	
subitem not at all important	○	○	◉	○	○	essential

Clear selection

Does your paper address subitem 1b-i? *

Copy and paste relevant sections from the manuscript abstract (include quotes in quotation marks "like this" to indicate direct quotes from your manuscript), or elaborate on this item by providing additional information not in the ms, or briefly explain why the item is not applicable/relevant for your study

"The intervention group received content incorporating various behavior change techniques designed to address key motivational and volitional determinants of hand hygiene behavior (e.g., skills, knowledge, intention, attitudes toward hand hygiene, risk perception, outcome expectancies, self-efficacy, action planning, coping planning, action control, and habit). In contrast, the active control group was exposed to behavior change techniques targeting only a subset of these determinants (i.e., skills, knowledge, and intention)."

1b-ii) Level of human involvement in the METHODS section of the ABSTRACT

Clarify the level of human involvement in the abstract, e.g., use phrases like “fully automated” vs. “therapist/nurse/care provider/physician-assisted” (mention number and expertise of providers involved, if any). (Note: Only report in the abstract what the main paper is reporting. If this information is missing from the main body of text, consider adding it)

	1	2	3	4	5	
subitem not at all important	○	●	○	○	○	essential

Clear selection

Does your paper address subitem 1b-ii?

Copy and paste relevant sections from the manuscript abstract (include quotes in quotation marks "like this" to indicate direct quotes from your manuscript), or elaborate on this item by providing additional information not in the ms, or briefly explain why the item is not applicable/relevant for your study

"The delivery of the intervention content was fully automated"

1b-iii) Open vs. closed, web-based (self-assessment) vs. face-to-face assessments in the METHODS section of the ABSTRACT

Mention how participants were recruited (online vs. offline), e.g., from an open access website or from a clinic or a closed online user group (closed usergroup trial), and clarify if this was a purely web-based trial, or there were face-to-face components (as part of the intervention or for assessment). Clearly say if outcomes were self-assessed through questionnaires (as common in web-based trials). Note: In traditional offline trials, an open trial (open-label trial) is a type of clinical trial in which both the researchers and participants know which treatment is being administered. To avoid confusion, use “blinded” or “unblinded” to indicated the level of blinding instead of “open”, as “open” in web-based trials usually refers to “open access” (i.e. participants can self-enrol). (Note: Only report in the abstract what the main paper is reporting. If this information is missing from the main body of text, consider adding it)

	1	2	3	4	5	
subitem not at all important	○	○	○	◉	○	essential

Clear selection

Does your paper address subitem 1b-iii?

Copy and paste relevant sections from the manuscript abstract (include quotes in quotation marks "like this" to indicate direct quotes from your manuscript), or elaborate on this item by providing additional information not in the ms, or briefly explain why the item is not applicable/relevant for your study

"We conducted a randomized controlled trial from March 2022 to April 2023 involving 193 participants who were recruited online" and "Hand hygiene was assessed with electronic diaries"

1b-iv) RESULTS section in abstract must contain use data

Report number of participants enrolled/assessed in each group, the use/uptake of the intervention (e.g., attrition/adherence metrics, use over time, number of logins etc.), in addition to primary/secondary outcomes. (Note: Only report in the abstract what the main paper is reporting. If this information is missing from the main body of text, consider adding it)

	1	2	3	4	5	
subitem not at all important	(selected)	○	○	○	○	essential

Clear selection

Does your paper address subitem 1b-iv?

Copy and paste relevant sections from the manuscript abstract (include quotes in quotation marks "like this" to indicate direct quotes from your manuscript), or elaborate on this item by providing additional information not in the ms, or briefly explain why the item is not applicable/relevant for your study

My answer

1b-v) CONCLUSIONS/DISCUSSION in abstract for negative trials

Conclusions/Discussions in abstract for negative trials: Discuss the primary outcome - if the trial is negative (primary outcome not changed), and the intervention was not used, discuss whether negative results are attributable to lack of uptake and discuss reasons. (Note: Only report in the abstract what the main paper is reporting. If this information is missing from the main body of text, consider adding it)

	1	2	3	4	5	
subitem not at all important	○	○	○	(selected)	○	essential

Clear selection

Does your paper address subitem 1b-v?

Copy and paste relevant sections from the manuscript abstract (include quotes in quotation marks "like this" to indicate direct quotes from your manuscript), or elaborate on this item by providing additional information not in the ms, or briefly explain why the item is not applicable/relevant for your study

"The randomized controlled trial provided evidence for the efficacy of Soapp+ to promote hand hygiene in the context of a pandemic"

INTRODUCTION

2a) In INTRODUCTION: Scientific background and explanation of rationale

2a-i) Problem and the type of system/solution

Describe the problem and the type of system/solution that is object of the study: intended as stand-alone intervention vs. incorporated in broader health care program? Intended for a particular patient population? Goals of the intervention, e.g., being more cost-effective to other interventions, replace or complement other solutions? (Note: Details about the intervention are provided in “Methods” under 5)

	1	2	3	4	5	
subitem not at all important	○	○	○	●	○	essential

Clear selection

Does your paper address subitem 2a-i? *

Copy and paste relevant sections from the manuscript (include quotes in quotation marks "like this" to indicate direct quotes from your manuscript), or elaborate on this item by providing additional information not in the ms, or briefly explain why the item is not applicable/relevant for your study

"During the early stages of the pandemic, we addressed the call for applying principles of behavior change to reduce the transmission of COVID-19 [2,3] and devised a multiphase optimization strategy (MOST) [6] to develop, optimize and evaluate a smartphone-based behavior change intervention to promote hand hygiene during the COVID-19 pandemic [7,8]. We opted for a digital intervention to avoid in-person interaction, which is crucial for preventing virus transmission. Additionally, digital interventions can be tailored to individual needs and have the potential to reach a large number of users. The MOST framework was deemed highly suitable for rigorously contextualizing the intervention to the pandemic through optimization. We targeted correct hand hygiene (i.e., hand washing or sanitizing) at key times because it represents an effective strategy in preventing the transmission of respiratory illnesses, including COVID-19 [9,10] and was therefore included in public health guidelines [11]."

2a-ii) Scientific background, rationale: What is known about the (type of) system

Scientific background, rationale: What is known about the (type of) system that is the object of the study (be sure to discuss the use of similar systems for other conditions/diagnoses, if appropiate), motivation for the study, i.e. what are the reasons for and what is the context for this specific study, from which stakeholder viewpoint is the study performed, potential impact of findings [2]. Briefly justify the choice of the comparator.

	1	2	3	4	5	
subitem not at all important	○	○	○	○	◉	essential

Clear selection

Does your paper address subitem 2a-ii? *

Copy and paste relevant sections from the manuscript (include quotes in quotation marks "like this" to indicate direct quotes from your manuscript), or elaborate on this item by providing additional information not in the ms, or briefly explain why the item is not applicable/relevant for your study

"The spread of the Coronavirus disease 2019 (COVID-19) pandemic represented an exceptional threat to human health worldwide. In that context, the collective adoption of protective behaviors (e.g., hand hygiene, physical distancing, mask wearing) became an essential measure to prevent the transmission of the virus, especially during the pandemic outbreak when there was no vaccine available [1,2]. Consequently, the development of effective behavior change interventions aiming at supporting the uptake and adoption of such behaviors emerged as public health priority [3]. However, during the course of the pandemic outbreak there was limited or no contextualized knowledge about how to effectively promote protective behaviors in the general population [4,5]"

2b) In INTRODUCTION: Specific objectives or hypotheses

Does your paper address CONSORT subitem 2b? *

Copy and paste relevant sections from the manuscript (include quotes in quotation marks "like this" to indicate direct quotes from your manuscript), or elaborate on this item by providing additional information not in the ms, or briefly explain why the item is not applicable/relevant for your study

"The current paper focuses on the evaluation phase of the MOST in which we tested the efficacy of Soapp+ (intervention group) by comparison to an active control group in a randomized controlled trial (RCT). As presented in the study protocol [7], we tested the following preregistered hypotheses:

- H1: The intervention group shows a greater increase in correct hand hygiene behavior at key times at the post-measure (H1a) and at 6-month follow-up (H1b) than the control group.
- H2: The intervention group shows a significant increase in the targeted behavioral determinants compared with the control group at the post-intervention measure (H2a) and at 6-month follow-up (H2b). Specifically, the target behavioral determinants were intention, attitude, risk perception, outcome expectancies, self-efficacy, action planning, coping planning, action control, and habit strength.

As secondary aim, we examined between group differences in flu-like infection symptoms and occurrences of Covid-19 (secondary outcomes)."

METHODS

3a) Description of trial design (such as parallel, factorial) including allocation ratio

Does your paper address CONSORT subitem 3a? *

Copy and paste relevant sections from the manuscript (include quotes in quotation marks "like this" to indicate direct quotes from your manuscript), or elaborate on this item by providing additional information not in the ms, or briefly explain why the item is not applicable/relevant for your study

"The study design for the evaluation phase was a double-blind RCT (1:1 ratio) comparing the optimized app Soapp+ with an active control group using a simplified version of the app that focused on providing information on hand hygiene."

3b) Important changes to methods after trial commencement (such as eligibility criteria), with reasons

Does your paper address CONSORT subitem 3b? *

Copy and paste relevant sections from the manuscript (include quotes in quotation marks "like this" to indicate direct quotes from your manuscript), or elaborate on this item by providing additional information not in the ms, or briefly explain why the item is not applicable/relevant for your study

This item is not applicable.

3b-i) Bug fixes, Downtimes, Content Changes

Bug fixes, Downtimes, Content Changes: ehealth systems are often dynamic systems. A description of changes to methods therefore also includes important changes made on the intervention or comparator during the trial (e.g., major bug fixes or changes in the functionality or content) (5-iii) and other “unexpected events” that may have influenced study design such as staff changes, system failures/downtimes, etc. [2].

	1	2	3	4	5	
subitem not at all important	○	○	◉	○	○	essential

Clear selection

Does your paper address subitem 3b-i?

Copy and paste relevant sections from the manuscript (include quotes in quotation marks "like this" to indicate direct quotes from your manuscript), or elaborate on this item by providing additional information not in the ms, or briefly explain why the item is not applicable/relevant for your study

This item is not applicable.

4a) Eligibility criteria for participants

Does your paper address CONSORT subitem 4a? *

Copy and paste relevant sections from the manuscript (include quotes in quotation marks "like this" to indicate direct quotes from your manuscript), or elaborate on this item by providing additional information not in the ms, or briefly explain why the item is not applicable/relevant for your study

"The target population were German speaking adults from the Swiss population interested in using an app to improve hand hygiene. Inclusion criteria were: i) being at least 18 years old, ii) owning a smartphone with mobile access to the internet, iii) being proficient in the German language, and iv) having signed an informed consent form to participate in the study."

4a-i) Computer / Internet literacy

Computer / Internet literacy is often an implicit “de facto” eligibility criterion - this should be explicitly clarified.

	1	2	3	4	5	
subitem not at all important	◉	○	○	○	○	essential

Clear selection

Does your paper address subitem 4a-i?

Copy and paste relevant sections from the manuscript (include quotes in quotation marks "like this" to indicate direct quotes from your manuscript), or elaborate on this item by providing additional information not in the ms, or briefly explain why the item is not applicable/relevant for your study

"Additionally, smartphone proficiency was an implicit eligibility criterion, as participants needed to perform various tasks on their smartphones prior to the study's commencement (e.g., downloading the app and registering)."

4a-ii) Open vs. closed, web-based vs. face-to-face assessments:

Open vs. closed, web-based vs. face-to-face assessments: Mention how participants were recruited (online vs. offline), e.g., from an open access website or from a clinic, and clarify if this was a purely web-based trial, or there were face-to-face components (as part of the intervention or for assessment), i.e., to what degree got the study team to know the participant. In online-only trials, clarify if participants were quasi-anonymous and whether having multiple identities was possible or whether technical or logistical measures (e.g., cookies, email confirmation, phone calls) were used to detect/prevent these.

	1	2	3	4	5	
subitem not at all important	○	○	○	◉	○	essential

Clear selection

Does your paper address subitem 4a-ii? *

Copy and paste relevant sections from the manuscript (include quotes in quotation marks "like this" to indicate direct quotes from your manuscript), or elaborate on this item by providing additional information not in the ms, or briefly explain why the item is not applicable/relevant for your study

"Participants were recruited via social media (e.g., Facebook, Instagram), mailing lists, and leaflets with the support of a market research company."

4a-iii) Information giving during recruitment

Information given during recruitment. Specify how participants were briefed for recruitment and in the informed consent procedures (e.g., publish the informed consent documentation as appendix, see also item X26), as this information may have an effect on user self-selection, user expectation and may also bias results.

	1	2	3	4	5	
subitem not at all important	○	○	○	◉	○	essential

Clear selection

Does your paper address subitem 4a-iii?

Copy and paste relevant sections from the manuscript (include quotes in quotation marks "like this" to indicate direct quotes from your manuscript), or elaborate on this item by providing additional information not in the ms, or briefly explain why the item is not applicable/relevant for your study

"Interested people were redirected to the study page on RedCap [29] where they could go through the study information, fill out an eligibility and consent survey, and sign the e-consent form. Afterwards, participants received a registration code via email and were guided to download the Soapp+ app from Apple App Store or Google Play Store and register to it."

4b) Settings and locations where the data were collected

Does your paper address CONSORT subitem 4b? *

Copy and paste relevant sections from the manuscript (include quotes in quotation marks "like this" to indicate direct quotes from your manuscript), or elaborate on this item by providing additional information not in the ms, or briefly explain why the item is not applicable/relevant for your study

"During the intervention phase, participants completed the hand hygiene diary on day 2, 8, 16, 24 and day 32." This item is less relevant as the data was collected through e-diaries embedded in the study app at various moments of the day; therefore, there was no specific location.

4b-i) Report if outcomes were (self-)assessed through online questionnaires

Clearly report if outcomes were (self-)assessed through online questionnaires (as common in web-based trials) or otherwise.

	1	2	3	4	5	
subitem not at all important	○	○	○	○	●	essential

Clear selection

Does your paper address subitem 4b-i? *

Copy and paste relevant sections from the manuscript (include quotes in quotation marks "like this" to indicate direct quotes from your manuscript), or elaborate on this item by providing additional information not in the ms, or briefly explain why the item is not applicable/relevant for your study

"The frequency of correct hand hygiene at key times was assessed using ecological momentary assessment with an electronic diary embedded in the study apps (i.e., Soapp+ and active control apps)."

4b-ii) Report how institutional affiliations are displayed

Report how institutional affiliations are displayed to potential participants [on ehealth media], as affiliations with prestigious hospitals or universities may affect volunteer rates, use, and reactions with regards to an intervention. (Not a required item – describe only if this may bias results)

	1	2	3	4	5	
subitem not at all important	(selected)	○	○	○	○	essential

Clear selection

Does your paper address subitem 4b-ii?

Copy and paste relevant sections from the manuscript (include quotes in quotation marks "like this" to indicate direct quotes from your manuscript), or elaborate on this item by providing additional information not in the ms, or briefly explain why the item is not applicable/relevant for your study

The institutional affiliations were reported on the flyiers and social media materials used during recruitment and on the study app. We don't think this item is relevant for our study as exposure to the institional information did not differ between intervention and active control groups.

5) The interventions for each group with sufficient details to allow replication, including how and when they were actually administered

5-i) Mention names, credential, affiliations of the developers, sponsors, and owners

Mention names, credential, affiliations of the developers, sponsors, and owners [6] (if authors/evaluators are owners or developer of the software, this needs to be declared in a "Conflict of interest" section or mentioned elsewhere in the manuscript).

	1	2	3	4	5	
subitem not at all important	○	●	○	○	○	essential

Clear selection

Does your paper address subitem 5-i?

Copy and paste relevant sections from the manuscript (include quotes in quotation marks "like this" to indicate direct quotes from your manuscript), or elaborate on this item by providing additional information not in the ms, or briefly explain why the item is not applicable/relevant for your study

"The Soapp+ app was developed by the study authors using technological resources provided by the Faculty of Human Sciences at the University of Bern, with support from an internal software engineer."

5-ii) Describe the history/development process

Describe the history/development process of the application and previous formative evaluations (e.g., focus groups, usability testing), as these will have an impact on adoption/use rates and help with interpreting results.

	1	2	3	4	5	
subitem not at all important	○	○	●	○	○	essential

Clear selection

Does your paper address subitem 5-ii?

Copy and paste relevant sections from the manuscript (include quotes in quotation marks "like this" to indicate direct quotes from your manuscript), or elaborate on this item by providing additional information not in the ms, or briefly explain why the item is not applicable/relevant for your study

This study focus on the evaluation stage of the Multiphase Optimization Strategy. Therefore, the app was alredy developed and optimized, going through various iterations.
"Following the MOST framework [12], the project included three phases: preparation, optimization, and evaluation. In the preparation phase [7], we developed a set of intervention modules and defined the optimization criteria. The development of intervention modules was guided by the Theoretical Domain Framework (TDF) [13]. First, we identified behavioral determinants of hand hygiene through a literature review, and through two focus group discussions with the target population [7]. Then, we mapped behavior change techniques (BCTs; Michie et al., 2013) to the behavioral determinants. Specifically, three intervention modules were developed targeting i) motivation, ii) habit formation and iii) social influence. The identified modules were paired and sequenced, resulting in nine different intervention conditions. Each intervention condition was hosted in the developed smartphone app Soapp. During the subsequent optimization phase, we devised a mixed-method double-blind parallel randomized trial between March and August 2021, which lasted 34 days. The trial aimed at identifying the intervention condition that met our optimization criteria: superiority regarding change in hand hygiene pre-post intervention, and regarding user satisfaction, usability, and engagement [8]. The results of the trial showed that hand hygiene improved while using the Soapp app. However, no superiority was found for any of the versions of the app. The qualitative results indicated that participants preferred the motivation and habit formation modules over the social influence one. A further emerging theme pointed out the need for better distribution of intervention content over time. Thus, in the optimized version of the Soapp app, named Soapp+, we selected the motivation and habit formation modules and delivered them in a parallel fashion rather than sequentially."

5-iii) Revisions and updating

Revisions and updating. Clearly mention the date and/or version number of the application/intervention (and comparator, if applicable) evaluated, or describe whether the intervention underwent major changes during the evaluation process, or whether the development and/or content was "frozen" during the trial. Describe dynamic components such as news feeds or changing content which may have an impact on the replicability of the intervention (for unexpected events see item 3b).

	1	2	3	4	5	
subitem not at all important	○	◉	○	○	○	essential

Clear selection

Does your paper address subitem 5-iii?

Copy and paste relevant sections from the manuscript (include quotes in quotation marks "like this" to indicate direct quotes from your manuscript), or elaborate on this item by providing additional information not in the ms, or briefly explain why the item is not applicable/relevant for your study

Not applicable - the intervention was never revised or updated.

5-iv) Quality assurance methods

Provide information on quality assurance methods to ensure accuracy and quality of information provided [1], if applicable.

	1	2	3	4	5	
subitem not at all important	◉	○	○	○	○	essential

Clear selection

Does your paper address subitem 5-iv?

Copy and paste relevant sections from the manuscript (include quotes in quotation marks "like this" to indicate direct quotes from your manuscript), or elaborate on this item by providing additional information not in the ms, or briefly explain why the item is not applicable/relevant for your study

Not applicable for this study.

5-v) Ensure replicability by publishing the source code, and/or providing screenshots/screen-capture video, and/or providing flowcharts of the algorithms used

Ensure replicability by publishing the source code, and/or providing screenshots/screen-capture video, and/or providing flowcharts of the algorithms used. Replicability (i.e., other researchers should in principle be able to replicate the study) is a hallmark of scientific reporting.

	1	2	3	4	5	
subitem not at all important	○	○	○	◉	○	essential

Clear selection

Does your paper address subitem 5-v?

Copy and paste relevant sections from the manuscript (include quotes in quotation marks "like this" to indicate direct quotes from your manuscript), or elaborate on this item by providing additional information not in the ms, or briefly explain why the item is not applicable/relevant for your study

Replicability for statistical analysis is ensured by making datasets and R code available on an Open Science Framework repository.

Replication for the content is ensured by reporting in the Multimedia Appendix 1 the intervention content delivered to the participants as well as the intervention timeline.

5-vi) Digital preservation

Digital preservation: Provide the URL of the application, but as the intervention is likely to change or disappear over the course of the years; also make sure the intervention is archived (Internet Archive, webcitation.org, and/or publishing the source code or screenshots/videos alongside the article). As pages behind login screens cannot be archived, consider creating demo pages which are accessible without login.

	1	2	3	4	5	
subitem not at all important	○	○	○	○	○	essential

Does your paper address subitem 5-vi?

Copy and paste relevant sections from the manuscript (include quotes in quotation marks "like this" to indicate direct quotes from your manuscript), or elaborate on this item by providing additional information not in the ms, or briefly explain why the item is not applicable/relevant for your study

My answer

5-vii) Access

Access: Describe how participants accessed the application, in what setting/context, if they had to pay (or were paid) or not, whether they had to be a member of specific group. If known, describe how participants obtained “access to the platform and Internet” [1]. To ensure access for editors/reviewers/readers, consider to provide a “backdoor” login account or demo mode for reviewers/readers to explore the application (also important for archiving purposes, see vi).

	1	2	3	4	5	
subitem not at all important	○	○	○	◉	○	essential

Clear selection

Does your paper address subitem 5-vii? *

Copy and paste relevant sections from the manuscript (include quotes in quotation marks "like this" to indicate direct quotes from your manuscript), or elaborate on this item by providing additional information not in the ms, or briefly explain why the item is not applicable/relevant for your study

"The content of the recruitment materials targeted individuals interested in forming new hand-hygiene habits and learning how to correctly wash or disinfect their hands to counter the spread of the virus. Interested people were redirected to the study page on RedCap [29] where they could go through the study information, fill out an eligibility and consent survey, and sign the e-consent form. Afterwards, participants received a registration code via email and were guided to download the Soapp+ app from Apple App Store or Google Play Store and register to it."

5-viii) Mode of delivery, features/functionalities/components of the intervention and comparator, and the theoretical framework

Describe mode of delivery, features/functionalities/components of the intervention and comparator, and the theoretical framework [6] used to design them (instructional strategy [1], behaviour change techniques, persuasive features, etc., see e.g., [7, 8] for terminology). This includes an in-depth description of the content (including where it is coming from and who developed it) [1],” whether [and how] it is tailored to individual circumstances and allows users to track their progress and receive feedback” [6]. This also includes a description of communication delivery channels and – if computer-mediated communication is a component – whether communication was synchronous or asynchronous [6]. It also includes information on presentation strategies [1], including page design principles, average amount of text on pages, presence of hyperlinks to other resources, etc. [1].

	1	2	3	4	5	
subitem not at all important	○	○	○	○	◉	essential

Clear selection

Does your paper address subitem 5-viii? *

Copy and paste relevant sections from the manuscript (include quotes in quotation marks "like this" to indicate direct quotes from your manuscript), or elaborate on this item by providing additional information not in the ms, or briefly explain why the item is not applicable/relevant for your study

Components of the intervention and comparator, and the theoretical framework used to design them (Behavior change techniques) si presented in the manuscript in Table 1 and as follow:
"The content of the intervention was delivered to participants via their personal smartphone through the study application Soapp+. The intervention group received the optimized Soapp+ app while the active control group was exposed to a simplified version of Soapp+. Table 1 shows the content delivered. The exact wording of the intervention messages and the intervention timeline can be found in the Multimedia Appendix 1, Table 1.3 and Figure 1.2.
Active control group. The active control group received information on how to perform correct hand hygiene (both in written and video formats), the key times to perform hand hygiene, and a statement of the goal of the app (basic module). This content was accessible throughout the intervention phase. Based on the results of the optimization phase, we also added a list of fun facts that were delivered to the participants via push notifications throughout the intervention phase. Finally, quizzes on hand hygiene aimed at ensuring that the active control group interacted with the app throughout the intervention phase.
Intervention group. Like the control group, the intervention group received the basic module. They additionally received the content targeting motivation and habit. Based on results of the optimization phase (i.e., request for better distributed content/tasks), this content was delivered in parallel instead of sequentially as done during the optimization trial."

Additionally, Multimedia Appenidix 1 contains a more detailed report of the intervention content (Table 1.3).

5-ix) Describe use parameters

Describe use parameters (e.g., intended “doses” and optimal timing for use). Clarify what instructions or recommendations were given to the user, e.g., regarding timing, frequency, heaviness of use, if any, or was the intervention used ad libitum.

	1	2	3	4	5	
subitem not at all important	○	○	○	○	○	essential

Does your paper address subitem 5-ix?

Copy and paste relevant sections from the manuscript (include quotes in quotation marks "like this" to indicate direct quotes from your manuscript), or elaborate on this item by providing additional information not in the ms, or briefly explain why the item is not applicable/relevant for your study

My answer

5-x) Clarify the level of human involvement

Clarify the level of human involvement (care providers or health professionals, also technical assistance) in the e-intervention or as co-intervention (detail number and expertise of professionals involved, if any, as well as "type of assistance offered, the timing and frequency of the support, how it is initiated, and the medium by which the assistance is delivered". It may be necessary to distinguish between the level of human involvement required for the trial, and the level of human involvement required for a routine application outside of a RCT setting (discuss under item 21 – generalizability).

	1	2	3	4	5	
subitem not at all important	○	○	○	○	○	essential

Does your paper address subitem 5-x?

Copy and paste relevant sections from the manuscript (include quotes in quotation marks "like this" to indicate direct quotes from your manuscript), or elaborate on this item by providing additional information not in the ms, or briefly explain why the item is not applicable/relevant for your study

My answer

5-xi) Report any prompts/reminders used

Report any prompts/reminders used: Clarify if there were prompts (letters, emails, phone calls, SMS) to use the application, what triggered them, frequency etc. It may be necessary to distinguish between the level of prompts/reminders required for the trial, and the level of prompts/reminders for a routine application outside of a RCT setting (discuss under item 21 – generalizability).

	1	2	3	4	5	
subitem not at all important	○	○	●	○	○	essential

Clear selection

Does your paper address subitem 5-xi? *

Copy and paste relevant sections from the manuscript (include quotes in quotation marks "like this" to indicate direct quotes from your manuscript), or elaborate on this item by providing additional information not in the ms, or briefly explain why the item is not applicable/relevant for your study

We did not send any ad-hoc prompt or reminder other than the ones that were scheduled by study design (as push notifications) to remind participants to fill out the diaries or to notify them as new content was available.

5-xii) Describe any co-interventions (incl. training/support)

Describe any co-interventions (incl. training/support): Clearly state any interventions that are provided in addition to the targeted eHealth intervention, as ehealth intervention may not be designed as stand-alone intervention. This includes training sessions and support [1]. It may be necessary to distinguish between the level of training required for the trial, and the level of training for a routine application outside of a RCT setting (discuss under item 21 – generalizability).

	1	2	3	4	5	
subitem not at all important	○	○	○	●	○	essential

Clear selection

Does your paper address subitem 5-xii? *

Copy and paste relevant sections from the manuscript (include quotes in quotation marks "like this" to indicate direct quotes from your manuscript), or elaborate on this item by providing additional information not in the ms, or briefly explain why the item is not applicable/relevant for your study

Not applicable - This was a stand alone intervention

6a) Completely defined pre-specified primary and secondary outcome measures, including how and when they were assessed

Does your paper address CONSORT subitem 6a? *

Copy and paste relevant sections from the manuscript (include quotes in quotation marks "like this" to indicate direct quotes from your manuscript), or elaborate on this item by providing additional information not in the ms, or briefly explain why the item is not applicable/relevant for your study

"Measures
Primary outcome
The frequency of correct hand hygiene at key times was assessed using ecological momentary assessment with an electronic diary embedded in the study apps (i.e., Soapp+ and active control apps). This approach was used to avoid retrospective bias in reporting hand hygiene [21]. On study days 2, 8, 16, 24, and 32, participants were prompted five times per day to indicate whether each of the 13 key times to perform hand hygiene defined by the Swiss Federal Office of Public Health occurred (e.g., arriving home, after using the toilet; see Multimedia Appendix 1, Table 1.2.). For each key situation that occurred, participants were asked how often they correctly washed or disinfected their hands. The 5-point response scale ranged from never (0) to always (4). For each diary day, the hand hygiene score was calculated by (1) computing the mean frequency of correct hand hygiene across all indicated key times in a single diary, and then (2) averaging the hand hygiene frequency across the five diaries.

Secondary outcomes
Targeted behavioral determinants. The following behavioral determinants were assessed at T1, T2, and follow-up. These variables were selected based on the results of the optimization phase and represented the determinants targeted by the Soapp+ intervention.

Behavioral intention. One self-reported item adapted from [22] asking: "To what extent do you intend to correctly perform your hand hygiene behavior at key times?". The response options ranged from 1 "not at all" to 6 "very strongly".

Action planning. Mean score of three self-reported items [23] such as: "I have made a detailed plan about when I am going to wash and disinfect my hands." The response options ranged from 1 "not at all" to 6 "very strongly." The scale was reliable (Cronbach's Alpha = .90 at T1).

Coping planning. Mean score of four self-reported items such as: "I have made a detailed plan about how I can perform correct hand hygiene behavior at key times, when soap and water are not available' The response options ranged from 1 "not at all" to 6 "very strongly". (Cronbach's Alpha = .84 at T1).

Habit strength. Assessed with the 'Self-Report Behavioural Automaticity Index' (SRBAI) [24] with four items such as: "Correct hand hygiene behavior at key times is something that I do automatically." The response options ranged from 1 "strongly disagree" to 7 "strongly agree". (Cronbach's Alpha = .93 at T1).

Action control. Mean score of three self-reported items such as "During the last two weeks I have constantly monitored myself whether I washed or disinfected my hands according to my plans" The response options were adapted from [25] and ranged from 1 "not at all" to 6 "very strongly". (Cronbach's Alpha = .91 at T1).

Self-efficacy. Mean score of eight self-reported items such as "How confident are you that you correctly wash/disinfect your hands at key times, when you are on the way" The response options ranged from 1 "not at all" to 6 "very strongly." Self-efficacy items were adapted from [23]. (Cronbach's Alpha = .91 at T1).

Attitude. Mean score of six self-reported bipolar items [26] introduced by the wording "Correct hand hygiene behavior at key times is..." and followed by pairs of bipolar adjectives (e.g., useless VS useful). (Cronbach's Alpha = .81 at T1).

Outcome expectancies. Mean score of eight self-reported items [27] such as "If I engage in correct hand hygiene, then then the risk drops that I contract pathogens". The response options ranged from 1 "this doesn't apply at all" to 6 "this applies very precisely". (Cronbach's Alpha = .72 at T1).

Risk perception. Mean score of five self-reported items [27] such as "to what extent do you think that you could contract corona, if you don't engage in correct hand hygiene in key situations?". The response options ranged from 1 "this doesn't apply at all" to 6 "this applies very precisely". (Cronbach's Alpha = .90 at T1).

Flu-like infection symptoms and occurrences of Covid-19. Participants reported on these secondary outcomes by answering the items "In the last two weeks, did you have flu-like infection symptoms?" and "In the last two weeks, did you have a positive corona test?".

Finally, social desirability was assessed at T1 using the German Short Scale for the Two-Dimensional Measurement of Social Desirability [28]"

6a-i) Online questionnaires: describe if they were validated for online use and apply CHERRIES items to describe how the questionnaires were designed/deployed

If outcomes were obtained through online questionnaires, describe if they were validated for online use and apply CHERRIES items to describe how the questionnaires were designed/deployed [9].

	1	2	3	4	5	
subitem not at all important	○	○	○	○	○	essential

Does your paper address subitem 6a-i?

Copy and paste relevant sections from manuscript text

My answer

6a-ii) Describe whether and how “use” (including intensity of use/dosage) was defined/measured/monitored

Describe whether and how “use” (including intensity of use/dosage) was defined/measured/monitored (logins, logfile analysis, etc.). Use/adoption metrics are important process outcomes that should be reported in any ehealth trial.

	1	2	3	4	5	
subitem not at all important	○	○	○	○	○	essential

Does your paper address subitem 6a-ii?

Copy and paste relevant sections from manuscript text

My answer

6a-iii) Describe whether, how, and when qualitative feedback from participants was obtained

Describe whether, how, and when qualitative feedback from participants was obtained (e.g., through emails, feedback forms, interviews, focus groups).

	1	2	3	4	5	
subitem not at all important	○	○	○	○	○	essential

Does your paper address subitem 6a-iii?

Copy and paste relevant sections from manuscript text

My answer

6b) Any changes to trial outcomes after the trial commenced, with reasons

Does your paper address CONSORT subitem 6b? *

Copy and paste relevant sections from the manuscript (include quotes in quotation marks "like this" to indicate direct quotes from your manuscript), or elaborate on this item by providing additional information not in the ms, or briefly explain why the item is not applicable/relevant for your study

Not applicable - there were no changes to trial outcomes after the trial commenced

7a) How sample size was determined

NPT: When applicable, details of whether and how the clustering by care provides or centers was addressed

7a-i) Describe whether and how expected attrition was taken into account when calculating the sample size

Describe whether and how expected attrition was taken into account when calculating the sample size.

	1	2	3	4	5	
subitem not at all important	○	○	○	●	○	essential

Clear selection

Does your paper address subitem 7a-i?

Copy and paste relevant sections from manuscript title (include quotes in quotation marks "like this" to indicate direct quotes from your manuscript), or elaborate on this item by providing additional information not in the ms, or briefly explain why the item is not applicable/relevant for your study

"The sample size was calculated a priori for an independent samples t-test (β= .80, α = .05, Cohen's d = 0.35). The target sample size was raised to 245 participants to account for a 20% dropout rate."

7b) When applicable, explanation of any interim analyses and stopping guidelines

Does your paper address CONSORT subitem 7b? *

Copy and paste relevant sections from the manuscript (include quotes in quotation marks "like this" to indicate direct quotes from your manuscript), or elaborate on this item by providing additional information not in the ms, or briefly explain why the item is not applicable/relevant for your study

We did not conduct any interim analysis.
"We stopped the trial 13 months after the start of the study due to the end of the project timeline. "

8a) Method used to generate the random allocation sequence
NPT: When applicable, how care providers were allocated to each trial group

Does your paper address CONSORT subitem 8a? *

Copy and paste relevant sections from the manuscript (include quotes in quotation marks "like this" to indicate direct quotes from your manuscript), or elaborate on this item by providing additional information not in the ms, or briefly explain why the item is not applicable/relevant for your study

"A simple randomization was implemented in Qualtrics, which preserved the allocation concealment."

8b) Type of randomisation; details of any restriction (such as blocking and block size)

Does your paper address CONSORT subitem 8b? *

Copy and paste relevant sections from the manuscript (include quotes in quotation marks "like this" to indicate direct quotes from your manuscript), or elaborate on this item by providing additional information not in the ms, or briefly explain why the item is not applicable/relevant for your study

"A simple randomization was implemented in Qualtrics, which preserved the allocation concealment. "

9) Mechanism used to implement the random allocation sequence (such as sequentially numbered containers), describing any steps taken to conceal the sequence until interventions were assigned

Does your paper address CONSORT subitem 9? *

Copy and paste relevant sections from the manuscript (include quotes in quotation marks "like this" to indicate direct quotes from your manuscript), or elaborate on this item by providing additional information not in the ms, or briefly explain why the item is not applicable/relevant for your study

"The day after the registration, participants received the baseline questionnaire (T1) and were then randomized to intervention or active control group. A simple randomization was implemented in Qualtrics, which preserved the allocation concealment. Additionally, the researchers involved in the study were blinded to intervention assignment because the participant identifier was pseudo-anonymized before randomization"

10) Who generated the random allocation sequence, who enrolled participants, and who assigned participants to interventions

Does your paper address CONSORT subitem 10? *

Copy and paste relevant sections from the manuscript (include quotes in quotation marks "like this" to indicate direct quotes from your manuscript), or elaborate on this item by providing additional information not in the ms, or briefly explain why the item is not applicable/relevant for your study

"Interested people were redirected to the study page on RedCap [29] where they could go through the study information, fill out an eligibility and consent survey, and sign the e-consent form. Afterwards, participants received a registration code via email and were guided to download the Soapp+ app from Apple App Store or Google Play Store and register to it. The day after the registration, participants received the baseline questionnaire (T1) and were then randomized to intervention or active control group. A simple randomization was implemented in Qualtrics, which preserved the allocation concealment."

11a) If done, who was blinded after assignment to interventions (for example, participants, care providers, those assessing outcomes) and how

NPT: Whether or not administering co-interventions were blinded to group assignment

11a-i) Specify who was blinded, and who wasn't

Specify who was blinded, and who wasn't. Usually, in web-based trials it is not possible to blind the participants [1, 3] (this should be clearly acknowledged), but it may be possible to blind outcome assessors, those doing data analysis or those administering co-interventions (if any).

	1	2	3	4	5	
subitem not at all important	○	○	○	◉	○	essential

Clear selection

Does your paper address subitem 11a-i? *

Copy and paste relevant sections from the manuscript (include quotes in quotation marks "like this" to indicate direct quotes from your manuscript), or elaborate on this item by providing additional information not in the ms, or briefly explain why the item is not applicable/relevant for your study

"The study design for the evaluation phase was a double-blind RCT (1:1 ratio) comparing the optimized app Soapp+ with an active control group using a simplified version of the app that focused on providing information on hand hygiene."

"Additionally, the researchers involved in the study were blinded to intervention assignment because the participant identifier was pseudo-anonymized before randomization"

11a-ii) Discuss e.g., whether participants knew which intervention was the "intervention of interest" and which one was the "comparator"

Informed consent procedures (4a-ii) can create biases and certain expectations - discuss e.g., whether participants knew which intervention was the "intervention of interest" and which one was the "comparator".

	1	2	3	4	5	
subitem not at all important	○	○	○	○	○	essential

Does your paper address subitem 11a-ii?

Copy and paste relevant sections from the manuscript (include quotes in quotation marks "like this" to indicate direct quotes from your manuscript), or elaborate on this item by providing additional information not in the ms, or briefly explain why the item is not applicable/relevant for your study

My answer

11b) If relevant, description of the similarity of interventions

(this item is usually not relevant for ehealth trials as it refers to similarity of a placebo or sham intervention to a active medication/intervention)

Does your paper address CONSORT subitem 11b? *

Copy and paste relevant sections from the manuscript (include quotes in quotation marks "like this" to indicate direct quotes from your manuscript), or elaborate on this item by providing additional information not in the ms, or briefly explain why the item is not applicable/relevant for your study

Not applicable as this is an ehealth trial. Differnces between intervention and active control groups are presented at point 5-VIII

12a) Statistical methods used to compare groups for primary and secondary outcomes

NPT: When applicable, details of whether and how the clustering by care providers or centers was addressed

Does your paper address CONSORT subitem 12a? *

Copy and paste relevant sections from the manuscript (include quotes in quotation marks "like this" to indicate direct quotes from your manuscript), or elaborate on this item by providing additional information not in the ms, or briefly explain why the item is not applicable/relevant for your study

"In order to test the hypotheses, the assessment points considered for hand hygiene behavior were the first diary at day 2 (T1), the diary day at day 32 (T2, post-intervention), and the last diary day at day 181 (follow-up). The difference in hand hygiene between T2 and T1 and follow-up and T1 was compared between the intervention and the active control group using the non-parametric Wilcoxon Rank Sum test. A positive value in hand hygiene difference over assessments indicated an increase in hand hygiene. Likewise, positive values in the targeted behavioral determinants over assessments indicated an increasing trend. The Wilcoxon Rank Sum test was chosen over the pre-registered Student’s t-test due to the non-normal distribution of the data, as assessed by the Shapiro-Wilk normality test, representing a deviation from the registered protocol."

12a-i) Imputation techniques to deal with attrition / missing values

Imputation techniques to deal with attrition / missing values: Not all participants will use the intervention/comparator as intended and attrition is typically high in ehealth trials. Specify how participants who did not use the application or dropped out from the trial were treated in the statistical analysis (a complete case analysis is strongly discouraged, and simple imputation techniques such as LOCF may also be problematic [4]).

	1	2	3	4	5	
subitem not at all important	○	○	○	○	◉	essential

Clear selection

Does your paper address subitem 12a-i? *

Copy and paste relevant sections from the manuscript (include quotes in quotation marks "like this" to indicate direct quotes from your manuscript), or elaborate on this item by providing additional information not in the ms, or briefly explain why the item is not applicable/relevant for your study

"To handle missing data the intention-to-treat (ITT) principle was adopted [30]. According to the ITT principle, all the randomized participants are included in the analysis, regardless of what happens after randomization (e.g., noncompliance, protocol deviations). Therefore, the ITT approach avoids overoptimistic estimates of the efficacy of an intervention resulting from the removal of noncompliers. Missing data and dropouts were addressed using the last observation carried forward approach (LOCF). LOCF represents a parsimonious yet effective method to maintained appropriate Type 1 error protection [31]. This approach has been used in previous behavior change research [e.g., 31], including the optimization trial of the Soapp app [8]. This methodological choice was reinforced by the results of a secondary analysis conducted on the optimization trial [33]. The analysis revealed a noteworthy intra-class correlation (ICC) of 0.60 in hand hygiene, signifying that a substantial portion of the observed behavior can be attributed to individual differences between persons. This implies that the behavior tends to be more consistent or less variable within individuals over time."

12b) Methods for additional analyses, such as subgroup analyses and adjusted analyses

Does your paper address CONSORT subitem 12b? *

Copy and paste relevant sections from the manuscript (include quotes in quotation marks "like this" to indicate direct quotes from your manuscript), or elaborate on this item by providing additional information not in the ms, or briefly explain why the item is not applicable/relevant for your study

"Finally, we also conducted a set of sensitivity analyses without missing value imputation; results are available in the Multimedia Appendix 1."

X26) REB/IRB Approval and Ethical Considerations [recommended as subheading under "Methods"] (not a CONSORT item)

X26-i) Comment on ethics committee approval

	1	2	3	4	5	
subitem not at all important	○	○	◉	○	○	essential

Clear selection

Does your paper address subitem X26-i?

Copy and paste relevant sections from the manuscript (include quotes in quotation marks "like this" to indicate direct quotes from your manuscript), or elaborate on this item by providing additional information not in the ms, or briefly explain why the item is not applicable/relevant for your study

"The trial received ethical approval from the Swiss Ethics Committee of the canton of Bern (ID: 2021-00164). The reporting of the trial is in line with the CONSORT guidelines [35] (Multimedia Appendix 2). Informed consent was obtained from all participants, who were also informed of their right to opt out at any stage of the study. To protect privacy and confidentiality, data were de-identified through pseudonymization. As compensation, participants had the opportunity to win one of three iPhone 12s as an incentive for their participation. Additionally, after completing T2, participants were offered a small gift (i.e., a bar of hand-soap and a thank you card) in order to prevent dropout, that was sent to their homes."

x26-ii) Outline informed consent procedures

Outline informed consent procedures e.g., if consent was obtained offline or online (how? Checkbox, etc.?), and what information was provided (see 4a-ii). See [6] for some items to be included in informed consent documents.

	1	2	3	4	5	
subitem not at all important	○	○	○	○	○	essential

Does your paper address subitem X26-ii?

Copy and paste relevant sections from the manuscript (include quotes in quotation marks "like this" to indicate direct quotes from your manuscript), or elaborate on this item by providing additional information not in the ms, or briefly explain why the item is not applicable/relevant for your study

My answer

X26-iii) Safety and security procedures

Safety and security procedures, incl. privacy considerations, and any steps taken to reduce the likelihood or detection of harm (e.g., education and training, availability of a hotline)

	1	2	3	4	5	
subitem not at all important	○	○	○	○	○	essential

Does your paper address subitem X26-iii?

Copy and paste relevant sections from the manuscript (include quotes in quotation marks "like this" to indicate direct quotes from your manuscript), or elaborate on this item by providing additional information not in the ms, or briefly explain why the item is not applicable/relevant for your study

My answer

RESULTS

13a) For each group, the numbers of participants who were randomly assigned, received intended treatment, and were analysed for the primary outcome
NPT: The number of care providers or centers performing the intervention in each group and the number of patients treated by each care provider in each center

Does your paper address CONSORT subitem 13a? *

Copy and paste relevant sections from the manuscript (include quotes in quotation marks "like this" to indicate direct quotes from your manuscript), or elaborate on this item by providing additional information not in the ms, or briefly explain why the item is not applicable/relevant for your study

"One hundred ninety-three participants were recruited and randomized into one of the two experimental conditions. Among these, 12 participants did not complete any of the six hand hygiene diary days while other 35 participants did not fill out the first diary at T1. Further three participants completed the first diary but did not encounter any of the key situations to perform hand hygiene during that day. These participants (n = 50) were excluded from the analysis because the main outcome (i.e., hand hygiene) at T1 was missing. Out of the 193 participants who were randomized, 146 (76%) filled out the hand hygiene diary at T1, 91 of these (47% of the randomized participants) completed the hand hygiene diary at T2, and 109 (56% of the randomized participants) completed the hand hygiene diary at the six-month follow-up. Figure 1 shows the participants' flow through randomization, T1 diary assessment, T3 diary assessment, and follow-up for each intervention group."

This section is complemented by a flow chart diagram (Figure 1)

13b) For each group, losses and exclusions after randomisation, together with reasons

Does your paper address CONSORT subitem 13b? (NOTE: Preferably, this is shown in a CONSORT flow diagram) *

Copy and paste relevant sections from the manuscript (include quotes in quotation marks "like this" to indicate direct quotes from your manuscript), or elaborate on this item by providing additional information not in the ms, or briefly explain why the item is not applicable/relevant for your study

"Among these, 12 participants did not complete any of the six hand hygiene diary days while other 35 participants did not fill out the first diary at T1. Further three participants completed the first diary but did not encounter any of the key situations to perform hand hygiene during that day. These participants (n = 50) were excluded from the analysis because the main outcome (i.e., hand hygiene) at T1 was missing. Out of the 193 participants who were randomized, 146 (76%) filled out the hand hygiene diary at T1, 91 of these (47% of the randomized participants) completed the hand hygiene diary at T2, and 109 (56% of the randomized participants) completed the hand hygiene diary at the six-month follow-up. Figure 1 shows the participants' flow through randomization, T1 diary assessment, T3 diary assessment, and follow-up for each intervention group."

This section is complemented by a flow chart diagram (Figure 1)

13b-i) Attrition diagram

Strongly recommended: An attrition diagram (e.g., proportion of participants still logging in or using the intervention/comparator in each group plotted over time, similar to a survival curve) or other figures or tables demonstrating usage/dose/engagement.

	1	2	3	4	5	
subitem not at all important	○	○	○	○	○	essential

Does your paper address subitem 13b-i?

Copy and paste relevant sections from the manuscript or cite the figure number if applicable (include quotes in quotation marks "like this" to indicate direct quotes from your manuscript), or elaborate on this item by providing additional information not in the ms, or briefly explain why the item is not applicable/relevant for your study

This information is provided in a flow chart diagram in Figure 1.

14a) Dates defining the periods of recruitment and follow-up

Does your paper address CONSORT subitem 14a? *

Copy and paste relevant sections from the manuscript (include quotes in quotation marks "like this" to indicate direct quotes from your manuscript), or elaborate on this item by providing additional information not in the ms, or briefly explain why the item is not applicable/relevant for your study

"The recruitment for the evaluation trial began on March 9th, 2022, and ended on October 10th, 2022. Follow-up data were collected between September 5th, 2022, and April 18th, 2023."

14a-i) Indicate if critical "secular events" fell into the study period

Indicate if critical "secular events" fell into the study period, e.g., significant changes in Internet resources available or "changes in computer hardware or Internet delivery resources"

	1	2	3	4	5	
subitem not at all important	○	○	○	○	○	essential

Does your paper address subitem 14a-i?

Copy and paste relevant sections from the manuscript (include quotes in quotation marks "like this" to indicate direct quotes from your manuscript), or elaborate on this item by providing additional information not in the ms, or briefly explain why the item is not applicable/relevant for your study

My answer

14b) Why the trial ended or was stopped (early)

Does your paper address CONSORT subitem 14b? *

Copy and paste relevant sections from the manuscript (include quotes in quotation marks "like this" to indicate direct quotes from your manuscript), or elaborate on this item by providing additional information not in the ms, or briefly explain why the item is not applicable/relevant for your study

"We stopped the trial 13 months after the start of the study due to the end of the project timeline."

15) A table showing baseline demographic and clinical characteristics for each group

NPT: When applicable, a description of care providers (case volume, qualification, expertise, etc.) and centers (volume) in each group

Does your paper address CONSORT subitem 15? *

Copy and paste relevant sections from the manuscript (include quotes in quotation marks "like this" to indicate direct quotes from your manuscript), or elaborate on this item by providing additional information not in the ms, or briefly explain why the item is not applicable/relevant for your study

"Socio-demographics and hand hygiene behavior at baseline are reported in Table 2 (see Table 1.1 in Multimedia Appendix 1 for comparison with the socio-demographic characteristics of the Swiss population). The figures refer to the 146 participants who completed the first diary at T1. Participants' mean age was 41 years, 69% were women, 70% had high-school qualifications, 46% were employed, and 24% were living alone. Descriptive statistics in hand hygiene behavior (mean = 3.05; median = 3.15; skewness = -1.24) suggested that hand hygiene behavior was already high at baseline and characterized by a moderate left tailed distribution. No significant differences in social desirability were found between the intervention and the control group (M1 = 4.72, M2 = 4.79, F(1, 189) = 0.44, P = .506)."

15-i) Report demographics associated with digital divide issues

In ehealth trials it is particularly important to report demographics associated with digital divide issues, such as age, education, gender, social-economic status, computer/Internet/ehealth literacy of the participants, if known.

	1	2	3	4	5	
subitem not at all important	○	○	○	○	○	essential

Does your paper address subitem 15-i? *

Copy and paste relevant sections from the manuscript (include quotes in quotation marks "like this" to indicate direct quotes from your manuscript), or elaborate on this item by providing additional information not in the ms, or briefly explain why the item is not applicable/relevant for your study

"Socio-demographics and hand hygiene behavior at baseline are reported in Table 2 (see Table 1.1 in Multimedia Appendix 1 for comparison with the socio-demographic characteristics of the Swiss population). The figures refer to the 146 participants who completed the first diary at T1. Participants' mean age was 41 years, 69% were women, 70% had high-school qualifications, 46% were employed, and 24% were living alone. Descriptive statistics in hand hygiene behavior (mean = 3.05; median = 3.15; skewness = -1.24) suggested that hand hygiene behavior was already high at baseline and characterized by a moderate left tailed distribution. No significant differences in social desirability were found between the intervention and the control group (M1 = 4.72, M2 = 4.79, F(1, 189) = 0.44, P = .506)."

16) For each group, number of participants (denominator) included in each analysis and whether the analysis was by original assigned groups

16-i) Report multiple “denominators” and provide definitions

Report multiple “denominators” and provide definitions: Report N’s (and effect sizes) “across a range of study participation [and use] thresholds” [1], e.g., N exposed, N consented, N used more than x times, N used more than y weeks, N participants “used” the intervention/comparator at specific pre-defined time points of interest (in absolute and relative numbers per group). Always clearly define “use” of the intervention.

	1	2	3	4	5	
subitem not at all important	○	○	○	○	○	essential

Does your paper address subitem 16-i? *

Copy and paste relevant sections from the manuscript (include quotes in quotation marks "like this" to indicate direct quotes from your manuscript), or elaborate on this item by providing additional information not in the ms, or briefly explain why the item is not applicable/relevant for your study

Descriptive statistics are reported with denominators and explicit percentages in Table 2.

16-ii) Primary analysis should be intent-to-treat

Primary analysis should be intent-to-treat, secondary analyses could include comparing only “users”, with the appropriate caveats that this is no longer a randomized sample (see 18-i).

	1	2	3	4	5	
subitem not at all important	○	○	○	○	◉	essential

Clear selection

Does your paper address subitem 16-ii?

Copy and paste relevant sections from the manuscript (include quotes in quotation marks "like this" to indicate direct quotes from your manuscript), or elaborate on this item by providing additional information not in the ms, or briefly explain why the item is not applicable/relevant for your study

"To handle missing data the intention-to-treat (ITT) principle was adopted [30]. According to the ITT principle, all the randomized participants are included in the analysis, regardless of what happens after randomization (e.g., noncompliance, protocol deviations). Therefore, the ITT approach avoids overoptimistic estimates of the efficacy of an intervention resulting from the removal of noncompliers."

17a) For each primary and secondary outcome, results for each group, and the estimated effect size and its precision (such as 95% confidence interval)

Does your paper address CONSORT subitem 17a? *

Copy and paste relevant sections from the manuscript (include quotes in quotation marks "like this" to indicate direct quotes from your manuscript), or elaborate on this item by providing additional information not in the ms, or briefly explain why the item is not applicable/relevant for your study

"Hand hygiene behavior at T1, T2 and follow-up is summarized in Table 3. From a descriptive point of view, the whole sample exhibited a non-significant increase in hand hygiene behavior from T1 (Median = 3.25, IQR = [2.63, 3.60]) to T2 (Median = 3.42, IQR = [2.62, 3.88]; W = 8904, P = .059, r = .11). This was followed by a significant decrease between T2 and the 6-month follow-up (Median = 3.25, IQR = [2.50, 3.65]; W = 11655, P = .040, r = .12). When looking at changes in hand hygiene within each experimental group, the data shows that hand hygiene between T1 and T2 remained almost stable for the active control group (W = 2050, P = .771, r = .03 while the intervention group showed a significant increase in hand hygiene after the intervention phase (W = 2386, P = .020, r = .19). At follow-up, both the active control group (W = 2313, P = .352, r = .08) and the intervention group (W = 2924, P = .677, r = .03) exhibited a non-significant decrease in hand hygiene compared to T1."

"The between group differences in hand hygiene behavior between T1 and T2 and T1 and follow-up are reported in Table 4. Non-parametric analysis with the Wilcoxon rank-sum test showed that the change in hand hygiene behavior between T1 and T2 (W=2034, P <.041, effect size r = .17) and between T1 and follow-up (W=2005, P <.031, effect size r = .18) significantly differed between the groups, indicating better hand hygiene in the intervention group."

17a-i) Presentation of process outcomes such as metrics of use and intensity of use

In addition to primary/secondary (clinical) outcomes, the presentation of process outcomes such as metrics of use and intensity of use (dose, exposure) and their operational definitions is critical. This does not only refer to metrics of attrition (13-b) (often a binary variable), but also to more continuous exposure metrics such as “average session length”. These must be accompanied by a technical description how a metric like a “session” is defined (e.g., timeout after idle time) [1] (report under item 6a).

	1	2	3	4	5	
subitem not at all important	○	○	○	○	○	essential

Does your paper address subitem 17a-i?

Copy and paste relevant sections from the manuscript (include quotes in quotation marks "like this" to indicate direct quotes from your manuscript), or elaborate on this item by providing additional information not in the ms, or briefly explain why the item is not applicable/relevant for your study

My answer

17b) For binary outcomes, presentation of both absolute and relative effect sizes is recommended

Does your paper address CONSORT subitem 17b? *

Copy and paste relevant sections from the manuscript (include quotes in quotation marks "like this" to indicate direct quotes from your manuscript), or elaborate on this item by providing additional information not in the ms, or briefly explain why the item is not applicable/relevant for your study

Not applicable - the outcome of the study is not binary.

18) Results of any other analyses performed, including subgroup analyses and adjusted analyses, distinguishing pre-specified from exploratory

Does your paper address CONSORT subitem 18? *

Copy and paste relevant sections from the manuscript (include quotes in quotation marks "like this" to indicate direct quotes from your manuscript), or elaborate on this item by providing additional information not in the ms, or briefly explain why the item is not applicable/relevant for your study

"All the hypotheses were also tested without applying any missing value imputation algorithm. Results are available in the supplementary materials (Multimedia Appendix 1) and confirm most of the findings. Additionally, Wilcoxon tests without missing values imputation showed between group differences in coping planning between T1 and T2, and in risk perception between T1 and follow-up in favor of the intervention group."

18-i) Subgroup analysis of comparing only users

A subgroup analysis of comparing only users is not uncommon in ehealth trials, but if done, it must be stressed that this is a self-selected sample and no longer an unbiased sample from a randomized trial (see 16-iii).

	1	2	3	4	5	
subitem not at all important	○	○	○	○	○	essential

Does your paper address subitem 18-i?

Copy and paste relevant sections from the manuscript (include quotes in quotation marks "like this" to indicate direct quotes from your manuscript), or elaborate on this item by providing additional information not in the ms, or briefly explain why the item is not applicable/relevant for your study

My answer

19) All important harms or unintended effects in each group

(for specific guidance see CONSORT for harms)

Does your paper address CONSORT subitem 19? *

Copy and paste relevant sections from the manuscript (include quotes in quotation marks "like this" to indicate direct quotes from your manuscript), or elaborate on this item by providing additional information not in the ms, or briefly explain why the item is not applicable/relevant for your study

Not applicable given the absence of risks in promoting hand hygiene as done in the current study.

19-i) Include privacy breaches, technical problems

Include privacy breaches, technical problems. This does not only include physical “harm” to participants, but also incidents such as perceived or real privacy breaches [1], technical problems, and other unexpected/unintended incidents. “Unintended effects” also includes unintended positive effects [2].

	1	2	3	4	5	
subitem not at all important	○	○	○	○	○	essential

Does your paper address subitem 19-i?

Copy and paste relevant sections from the manuscript (include quotes in quotation marks "like this" to indicate direct quotes from your manuscript), or elaborate on this item by providing additional information not in the ms, or briefly explain why the item is not applicable/relevant for your study

My answer

19-ii) Include qualitative feedback from participants or observations from staff/researchers

Include qualitative feedback from participants or observations from staff/researchers, if available, on strengths and shortcomings of the application, especially if they point to unintended/unexpected effects or uses. This includes (if available) reasons for why people did or did not use the application as intended by the developers.

	1	2	3	4	5	
subitem not at all important	○	○	○	○	○	essential

Does your paper address subitem 19-ii?

Copy and paste relevant sections from the manuscript (include quotes in quotation marks "like this" to indicate direct quotes from your manuscript), or elaborate on this item by providing additional information not in the ms, or briefly explain why the item is not applicable/relevant for your study

My answer

DISCUSSION

22) Interpretation consistent with results, balancing benefits and harms, and considering other relevant evidence

NPT: In addition, take into account the choice of the comparator, lack of or partial blinding, and unequal expertise of care providers or centers in each group

22-i) Restate study questions and summarize the answers suggested by the data, starting with primary outcomes and process outcomes (use)

Restate study questions and summarize the answers suggested by the data, starting with primary outcomes and process outcomes (use).

	1	2	3	4	5	
subitem not at all important	○	○	○	○	○	essential

Does your paper address subitem 22-i? *

Copy and paste relevant sections from the manuscript (include quotes in quotation marks "like this" to indicate direct quotes from your manuscript), or elaborate on this item by providing additional information not in the ms, or briefly explain why the item is not applicable/relevant for your study

"As part of a MOST to develop, optimize, and test a smartphone-based hand hygiene intervention during the COVID-19 pandemic, this RCT aimed at evaluating the efficacy of the Soapp+ app intervention against an active control group. Results showed that hand hygiene can be increased short and long-term using the Soapp+ app intervention compared to active controls towards the end of the COVID-19 pandemic. Specifically, we observed small but stable effect sizes (r = 0.17 between pre-post intervention, and r = 0.18 between pre-intervention and follow-up) for increase in hand hygiene among the intervention group compared to the control group. The current results contribute to the notion that simultaneously targeting both reflective and automatic processes is an effective strategy for promoting and sustaining behavior change, even in challenging contexts such as a pandemic."

"Additionally, even though between-group differences in hand hygiene were small, we saw a trend that they resulted in fewer self-reported flu-like symptoms at post-intervention and follow-up for the intervention group (Multimedia Appendix 1, table 2.5). Although this difference was not statistically significant due to the small sample size and claims about the clinical significance of the intervention cannot be made, it does indicate a tendency toward an intervention effect on flu-like symptoms."

"Regarding our second hypothesis, results evidenced significant group differences pre-post intervention in coping planning in favor of the intervention group (see table 4)."

22-ii) Highlight unanswered new questions, suggest future research

Highlight unanswered new questions, suggest future research.

	1	2	3	4	5	
subitem not at all important	○	○	○	○	○	essential

Does your paper address subitem 22-ii?

Copy and paste relevant sections from the manuscript (include quotes in quotation marks "like this" to indicate direct quotes from your manuscript), or elaborate on this item by providing additional information not in the ms, or briefly explain why the item is not applicable/relevant for your study

"A key practical implication is that the Soapp+ app, a behavior change intervention contextualized to a pandemic, is now available and can be readily offered to the general public at the emergence of a future infectious disease outbreak (e.g., via public health institutions). However, since the app's efficacy has not yet been robustly demonstrated due to sample size, we recommend conducting a larger-scale RCT in a future outbreak. It is also important to note that testing behavior change interventions like Soapp+ during a pandemic remains challenging due to the constantly evolving context, including shifts in the pandemic trajectory, public health policies, government regulations, and medical advancements such as vaccine availability. These multifaceted and evolving factors likely contributed to the observed temporal variations in the adoption of hand hygiene behaviors, with implications for the evaluation of behavior change interventions [33,38]. For this reason, we believe that further testing of Soapp+ could extend beyond pandemic contexts and be conducted in more feasible settings, provided that the BCTs specifically referring to the Covid-19 pandemic (e.g., the ones targeting risk perception and outcome expectancies, see Table 1.3 in Multimedia Appendix 1) are adapted to the specific characteristics of the new context. Upon confirmation of the current positive effect of Soapp+ on hand hygiene, this further testing beyond the pandemic context would also pave the way for a broader application of Soapp+, particularly in settings where there is a demand for hand hygiene interventions (e.g., healthcare settings, during regular flu seasons, and to prevent the transmission of other viruses)."

20) Trial limitations, addressing sources of potential bias, imprecision, and, if relevant, multiplicity of analyses

20-i) Typical limitations in ehealth trials

Typical limitations in ehealth trials: Participants in ehealth trials are rarely blinded. Ehealth trials often look at a multiplicity of outcomes, increasing risk for a Type I error. Discuss biases due to non-use of the intervention/usability issues, biases through informed consent procedures, unexpected events.

	1	2	3	4	5	
subitem not at all important	○	○	○	○	○	essential

Does your paper address subitem 20-i? *

Copy and paste relevant sections from the manuscript (include quotes in quotation marks "like this" to indicate direct quotes from your manuscript), or elaborate on this item by providing additional information not in the ms, or briefly explain why the item is not applicable/relevant for your study

"This study is not without limitations. First, the achieved sample size (N = 143) was smaller than planned. Therefore, the probability of detecting a true effect of the intervention was below standard (i.e., .80) and some small effect sizes might have been missed. The decision to stop the recruitment even though the target sample size was not reached was due to the project timeline [7], but also considering that the pandemic situation was developing towards better scenarios till the point where the WHO declared the end of the global health emergency [20]. Based on the found effect size, future efficacy trials should be powered to detect small effects.
A second limitation is related to the dropouts between randomization and the first diary assessment. Indeed, the first diary was scheduled for the day after the randomization and some participants who had been randomized (n = 47) did not complete it. Other participants dropped out through the course of the study without filling out the assessment at T2 and follow-up. A possible explanation for the attrition might be due to the longitudinal study design with multiple diaries and other daily tasks that might have generated some fatigue. To address potential differences between dropouts and retainers, we conducted a dropout analysis, indicating a higher likelihood of younger participants dropping out. Thus, when extending the current results to young adults, caution is advised. "

21) Generalisability (external validity, applicability) of the trial findings

NPT: External validity of the trial findings according to the intervention, comparators, patients, and care providers or centers involved in the trial

21-i) Generalizability to other populations

Generalizability to other populations: In particular, discuss generalizability to a general Internet population, outside of a RCT setting, and general patient population, including applicability of the study results for other organizations

	1	2	3	4	5	
subitem not at all important	○	○	○	○	○	essential

Does your paper address subitem 21-i?

Copy and paste relevant sections from the manuscript (include quotes in quotation marks "like this" to indicate direct quotes from your manuscript), or elaborate on this item by providing additional information not in the ms, or briefly explain why the item is not applicable/relevant for your study

My answer

21-ii) Discuss if there were elements in the RCT that would be different in a routine application setting

Discuss if there were elements in the RCT that would be different in a routine application setting (e.g., prompts/reminders, more human involvement, training sessions or other co-interventions) and what impact the omission of these elements could have on use, adoption, or outcomes if the intervention is applied outside of a RCT setting.

	1	2	3	4	5	
subitem not at all important	○	○	○	○	○	essential

Does your paper address subitem 21-ii?

Copy and paste relevant sections from the manuscript (include quotes in quotation marks "like this" to indicate direct quotes from your manuscript), or elaborate on this item by providing additional information not in the ms, or briefly explain why the item is not applicable/relevant for your study

My answer

OTHER INFORMATION

23) Registration number and name of trial registry

Does your paper address CONSORT subitem 23? *

Copy and paste relevant sections from the manuscript (include quotes in quotation marks "like this" to indicate direct quotes from your manuscript), or elaborate on this item by providing additional information not in the ms, or briefly explain why the item is not applicable/relevant for your study

"ClinicalTrials.gov: NCT04830761"

24) Where the full trial protocol can be accessed, if available

Does your paper address CONSORT subitem 24? *

Cite a Multimedia Appendix, other reference, or copy and paste relevant sections from the manuscript (include quotes in quotation marks "like this" to indicate direct quotes from your manuscript), or elaborate on this item by providing additional information not in the ms, or briefly explain why the item is not applicable/relevant for your study

The full trial protocol is mentioned in the text and is part of the referneces:
[7] Amrein MA, Ruschetti GG, Baeder C, Bamert M, Inauen J. Mobile intervention to promote correct hand hygiene at key times to prevent COVID-19 in the Swiss adult general population: study protocol of a multiphase optimisation strategy. BMJ Open British Medical Journal Publishing Group; 2022 Mar 1;12(3):e055971. PMID:35351716

25) Sources of funding and other support (such as supply of drugs), role of funders

Does your paper address CONSORT subitem 25? *

Copy and paste relevant sections from the manuscript (include quotes in quotation marks "like this" to indicate direct quotes from your manuscript), or elaborate on this item by providing additional information not in the ms, or briefly explain why the item is not applicable/relevant for your study

"Funding: Ursula-Wirz Stiftung"

X27) Conflicts of Interest (not a CONSORT item)

X27-i) State the relation of the study team towards the system being evaluated

In addition to the usual declaration of interests (financial or otherwise), also state the relation of the study team towards the system being evaluated, i.e., state if the authors/evaluators are distinct from or identical with the developers/sponsors of the intervention.

	1	2	3	4	5	
subitem not at all important	○	○	○	○	○	essential

Does your paper address subitem X27-i?

Copy and paste relevant sections from the manuscript (include quotes in quotation marks "like this" to indicate direct quotes from your manuscript), or elaborate on this item by providing additional information not in the ms, or briefly explain why the item is not applicable/relevant for your study

My answer

About the CONSORT EHEALTH checklist

As a result of using this checklist, did you make changes in your manuscript? *

- ○ yes, major changes
- ◉ yes, minor changes
- ○ no

What were the most important changes you made as a result of using this checklist?

My answer

How much time did you spend on going through the checklist INCLUDING making changes in your manuscript *

A total of 6 hours (including making changes in the manuscript)

As a result of using this checklist, do you think your manuscript has improved? *

- (x) yes
- ( ) no
- ( ) Other:

Would you like to become involved in the CONSORT EHEALTH group?

This would involve for example becoming involved in participating in a workshop and writing an "Explanation and Elaboration" document

- ( ) yes
- ( ) no
- ( ) Other:

Any other comments or questions on CONSORT EHEALTH

My answer

STOP - Save this form as PDF before you click submit

To generate a record that you filled in this form, we recommend to generate a PDF of this page (on a Mac, simply select "print" and then select "print as PDF") before you submit it.

When you submit your (revised) paper to JMIR, please upload the PDF as supplementary file.

Don't worry if some text in the textboxes is cut off, as we still have the complete information in our database. Thank you!

Final step: Click submit !

Click submit so we have your answers in our database!

Send

Delete all entries

Never share passwords through Google Forms.

This content was not created by Google and is not endorsed by Google. Report Abuse - Terms of Use - Privacy Policy

Google Forms
